# The Association between Blood Test Trends and Undiagnosed Cancer: A Systematic Review and Critical Appraisal

**DOI:** 10.3390/cancers16091692

**Published:** 2024-04-26

**Authors:** Pradeep S. Virdee, Kiana K. Collins, Claire Friedemann Smith, Xin Yang, Sufen Zhu, Sophie E. Roberts, Nia Roberts, Jason L. Oke, Clare Bankhead, Rafael Perera, FD Richard Hobbs, Brian D. Nicholson

**Affiliations:** 1 Nuffield Department of Primary Care Health Sciences, Radcliffe Observatory Quarter, University of Oxford, Woodstock Road, Oxford OX2 6GG, UK; kiana.collins@st-hughs.ox.ac.uk (K.K.C.); claire.friedemann@phc.ox.ac.uk (C.F.S.); sufen.zhu@phc.ox.ac.uk (S.Z.); jason.oke@phc.ox.ac.uk (J.L.O.); clare.bankhead@phc.ox.ac.uk (C.B.); rafael.perera@phc.ox.ac.uk (R.P.); richard.hobbs@phc.ox.ac.uk (F.R.H.); brian.nicholson@phc.ox.ac.uk (B.D.N.); 2 St Edmund Hall, University of Oxford, Oxford OX1 4AR, UK; xin.yang@seh.ox.ac.uk; 3 Medical Sciences Division, St Peters College, University of Oxford, Oxford OX1 2DL, UK; sophie.roberts@spc.ox.ac.uk; 4 Bodleian Health Care Libraries, Oxford OX3 7DQ, UK; nia.roberts@bodleian.ox.ac.uk

**Keywords:** blood test, hematologic tests, trend, primary health care, cancer, neoplasms, systematic review

## Abstract

**Simple Summary:**

Blood tests are commonly requested by doctors.Blood tests are done for many reasons and invesitgating symptoms or monitoring existing conditions are just examples. For instance, blood tests can also be done as part of a routine health checkup to investigate symptoms or monitor existing conditions. For instance, blood tests can also be done as part of a routine health checkup. Some clinical guidelines for doctors include recommendations to investigate for cancer if a blood test indicates results that are lower or higher than normal levels. These recommendations are only helpful for a small number of cancers, such as bowel or pancreatic cancers. A patient can have repeated blood tests, allowing doctors to monitor how blood test results change over time. These changes over time are referred to as ‘trends’ and may provide doctors with more information than the results of a single blood test. For example, noting a small drop in a steady trend could be more useful than waiting for the blood test to drop below a normal level. Until now, there has been no research regarding whether blood test trends can identify patients who should be investigated for cancer. We aimed to assess this possibility in this research project.

**Abstract:**

Clinical guidelines include monitoring blood test abnormalities to identify patients at increased risk of undiagnosed cancer. Noting blood test changes over time may improve cancer risk stratification by considering a patient’s individual baseline and important changes within the normal range. We aimed to review the published literature to understand the association between blood test trends and undiagnosed cancer. MEDLINE and EMBASE were searched until 15 May 2023 for studies assessing the association between blood test trends and undiagnosed cancer. We used descriptive summaries and narratively synthesised studies. We included 29 articles. Common blood tests were haemoglobin (24%, n = 7), C-reactive protein (17%, n = 5), and fasting blood glucose (17%, n = 5), and common cancers were pancreatic (29%, n = 8) and colorectal (17%, n = 5). Of the 30 blood tests studied, an increasing trend in eight (27%) was associated with eight cancer types, and a decreasing trend in 17 (57%) with 10 cancer types. No association was reported between trends in 11 (37%) tests and breast, bile duct, glioma, haematological combined, liver, prostate, or thyroid cancers. Our review highlights trends in blood tests that could facilitate the identification of individuals at increased risk of undiagnosed cancer. For most possible combinations of tests and cancers, there was limited or no evidence.

## 1. Background

One in two patients are diagnosed with cancer in the UK [1]. Almost half (45.5%) of cases are diagnosed in the late stage (stage 3 and 4) [2], when likelihood of survival is poor [3,4,5,6]. Earlier detection can increase the likelihood of successful treatment and therefore improve patient outcomes and reduce cancer-related mortality [7]. Screening programmes may contribute to early detection but have been implemented only for a minority of cancers [8]. Most cancers are diagnosed following referral from primary care providers for symptoms that could represent undiagnosed cancer [9].

Blood tests commonly available in clinical practice, such as the full blood count and liver function tests, have an important role in the risk stratification of symptomatic patients in primary care for cancer investigation [10,11,12,13,14]. Blood tests are almost entirely considered in isolation (without repeat tests) under current clinical guidance, except in the management of intervention or disease [14]. Individual cancer risk associated with abnormalities in most common blood tests is too low to warrant urgent cancer investigation, except for anaemia, for colorectal cancer, and jaundice, for pancreatic cancer [15]. For all cancers combined, blood test abnormalities known to increase the risk of cancer above the 3% threshold recommend by the National Institute for Health and Care Excellence (NICE) for urgent investigation are low albumin, raised platelets, raised calcium, and raised inflammatory markers [14]. Although these abnormalities give general practitioners (GPs) an indication of potential underlying cancer, they allow uncertainty over which cancer(s) should be investigated.

Incorporating trends (changes over time) in repeated blood tests may provide more reliable risk stratification for cancer in general and for individual cancer sites [16]. For example, a patient with a low-normal haemoglobin may not be considered high-risk if the haemoglobin result is interpreted in isolation and a binary threshold for abnormality is used, but a low-normal result following years of high-normal results may represent an opportunity for cancer investigation. Incorporating such trends over repeated blood tests may hold greater potential to rule-in and rule-out referral for cancer investigation, although this potential remains unrealised.

We aimed to conduct a systematic review of the current literature reporting the association between trends in blood tests that are commonly used in primary care and undiagnosed cancer. This review aims to set a foundation for future research on the utility of blood test trends to facilitate cancer detection.

## 2. Methods

We followed the Preferred Reporting Items for Systematic review and Meta-Analysis (PRISMA) guidelines for the reporting of this review [17]. Ethical approval was not required, as there were no direct patient investigations in this study, and only published articles were systematically reviewed. The review protocol was registered with the International Prospective Register of Systematic Reviews (PROSPERO) database on 25 July 2022 (CRD42022348907).

### 2.1. Participants

We included studies of human participants aged 18 years or older reporting the association between trends in blood tests commonly available in clinical practice and cancer diagnosis in any clinical setting. We excluded blood tests taken after cancer diagnosis to predict prognosis or to monitor treatment.

### 2.2. Outcome

The main outcome was a first diagnosis of cancer across all cancer sites, including composite cancer sub-groupings and all cancers combined.

### 2.3. Search Strategy

The MEDLINE (OVID) [1946–present] and EMBASE (OVID) [1974–present] databases were searched from inception to 15 May 2023 to identify articles reporting on the association between trends in blood tests and a cancer diagnosis. The initial search was conducted in June 2022, with a full update in February and May 2023. Search terms included MeSH headings and title, abstract, and author keywords for blood tests, cancer diagnosis, and prediction or risk. No language or other limits were applied to the search. The full search strategy for each database is provided in Table S1 (MEDLINE) and Table S2 (EMBASE). In the eligible studies, we actively searched through each article’s reference list to find eligible studies that were not identified by the search strategy.

### 2.4. Study Selection

The full reference set initially underwent de-duplication in Endnote 20 [18] (by N.R.). Abstract and title screening were performed in Endnote 20 and Rayyan [19] (by P.S.V., K.K.C., C.F.S., X.Y.). Full-text screening was subsequently performed (by P.S.V.) to identify the study sample for data extraction and analysis. We included primary research articles reporting the association between trends in at least one blood test parameter (Table 1) and the subsequent diagnosis of cancer.

We excluded abstracts and conference proceedings, as they produce incomplete data for a thorough review. Studies using a cross-sectional design were excluded, as the data reflects a ‘snapshot’ at a certain time and hence, cannot identify trends or assess risk. Clinical trials of treatment intervention were excluded to reduce the influence of treatments on blood test data. Existing systematic reviews, correspondence, and case studies pertaining to <5 individuals were excluded. Non-English full-texts without English versions available or non-translatable were excluded.

### 2.5. Data Extraction

Data were extracted using an extraction form in Microsoft Excel. Data items included study design and population, blood test details, analytic methods and findings (such as effect estimates), cancer site, etc. The form was piloted on three randomly selected eligible articles. Data extraction for each eligible article was performed by two reviewers independently (P.S.V., K.K.C., C.F.S., X.Y., S.Z., S.E.R.), with discrepancies discussed until agreement was reached.

### 2.6. Data Analysis and Synthesis

Quantitative data were summarised using means with standard deviations (SD) for continuous data and counts with proportions for categorical data. We planned a random-effects meta-analysis of effect estimates, but due to the large variation in analytic methods used per blood test, this was not possible. Alternatively, we narratively described and critically appraised the findings. Narrative summaries included the definition of the blood test trend, analysis strategies, and findings.

### 2.7. Risk of Bias Assessment

Risk of bias was assessed in each study using the Quality In Prognosis Studies (QUIPS) tool [20]. Each study was assessed by two reviewers independently (P.S.V., K.K.C., C.F.S., X.Y., S.Z., S.E.R.), with discrepancies discussed until agreement was reached.

## 3. Results

**Figure 1:**
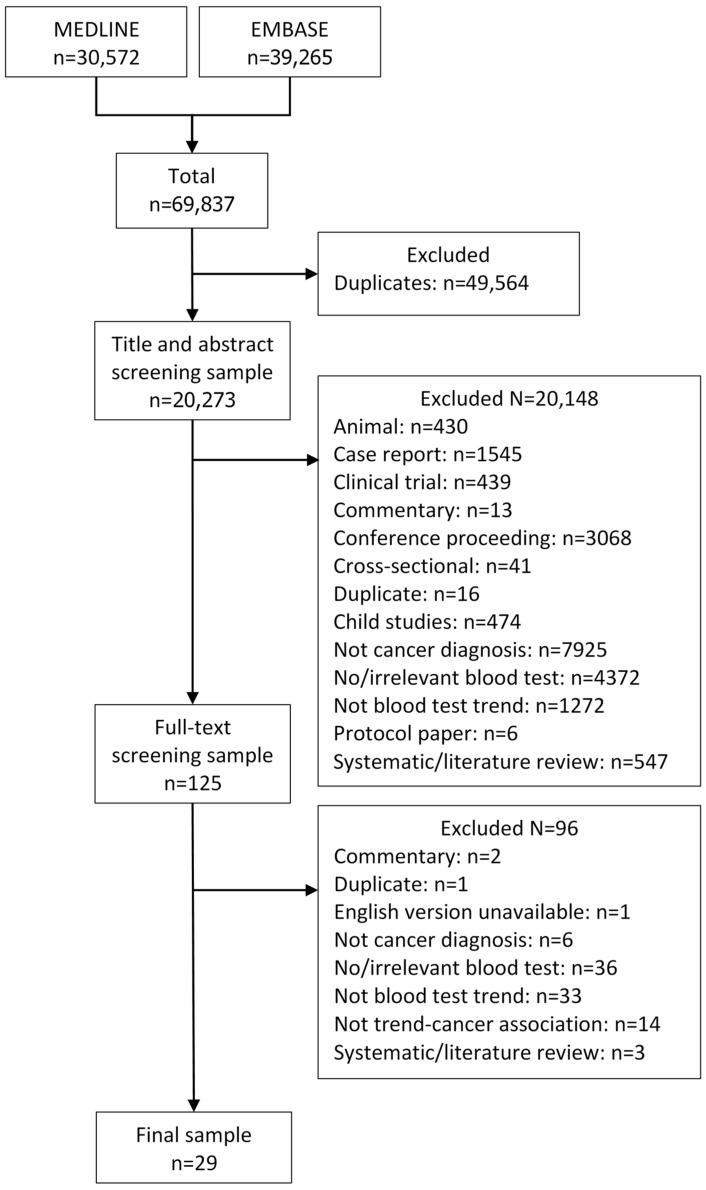
PRISMA diagram.

In total, 69,837 references were identified, of which 20,273 were unique after de-duplication (Figure 1). A total of 29 studies met the eligibility criteria and were included in the review [21,22,23,24,25,26,27,28,29,30,31,32,33,34,35,36,37,38,39,40,41,42,43,44,45,46,47,48,49].

### 3.1. Description of Studies

#### 3.1.1. Study Design

A description of each study is provided in Table S3. Of the 29 studies, a case–control design was used by 65% (n = 19), a cohort design by 21% (n = 6), and a case series by 14% (n = 4). Retrospective datasets were used by 83% (n = 24), prospectively collected data by 14% (n = 4), and the type was unclear in one study (Furukawa 1984 [27]). Data were collected from electronic health record databases by 65% (n = 19), clinical centres by 14% (n = 4), both by 14% (n = 4), among employees at a printing company in one study (Kubo 2016 [38]), and the source was unclear in one study (Furukawa 1984 [27]). The reason for blood testing was to screen for cancer in 24% (n = 7) of studies, whereas 76% (n = 22) used opportunistic tests (i.e., performed for any reason, excluding screening for cancer, such as to monitor symptoms or co-morbidity).

#### 3.1.2. Participants and Setting

The mean number of participants recruited was 1099 among prospective studies and 76,579 among retrospective studies, ranging from 9 to 939,949 participants over all the studies. The 29 articles spanned 12 different countries, with most studies being conducted in the USA (28%, n = 8) and UK (21%, n = 6). The period of recruitment ranged from 1968 to 2022. A total of 41% (n = 12) of studies were conducted in primary care, 14% (n = 4) in secondary care, and 21% (n = 6) in other settings, including: one study each in regards to blood donors, a specific population, postmenopausal women, pregnant women, a printing company, and a screening population. The setting was unclear in 24% (n = 7). Across the 18 studies that reported age, the mean age was 64.6 years (SD = 8.7). Across the 24 studies that described sex, 51.1% (SD = 24.0) of participants included were female.

### 3.2. Modelling Blood Test Trend

A total of 30 blood tests were assessed (Tables S4 and S5), with haemoglobin (24%, n = 7 studies) [21,25,29,37,40,46,49], C-reactive protein (17%, n = 5) [23,30,37,47,48], and fasting blood glucose (17%, n = 5) [26,33,35,41,44] most commonly studied (Figure S1). Most studies analysed blood test trends measured up to diagnosis (67%, n = 19). Trends were derived using three repeated blood tests, on average, among the 11 (38%) studies that reported this data (Table S6). The period over which repeated blood tests were obtained ranged from 30 days to 15 years.

To address missing blood test data, 24% (n = 7) of studies performed a complete case analysis [22,30,31,35,43,47,48], 3% (n = 1) derived missing blood levels from other available blood levels using known mathematical relationships (e.g., MCH = Hb/RBC and basophil% = basophil#/WBC) [49], 7% (n = 2) used imputation methods [39,46], and 3% (n = 1) used other methods [26]. Methods for handling missing blood test data were not discussed in 59% (n = 17) of studies. One study indicated there were no missing data [38].

During analysis, 93% (n = 27) studies analysed the trend as a continuous variable and 7% (n = 2) categorised the trend [27,48]. Furukawa 1984 [27] categorised aspartate transaminase trend into three groups, based on the pattern of change over time: fluctuating trend over >20 ng/mL (group 1), spike shaped rise, but mostly <20 ng/mL (group 2), and <20 ng/mL throughout (group 3) and used t-tests between groups. Toriola 2011 [48] categorised C-reactive protein trend into three groups: <1.1 mg/L (group 1), 1.1 to ≤2.6 mg/L (group 2), and >2.6 mg/L (group 3) and used logistic regression to analyse categorised trends. Of the studies which analysed the trend as a continuous variable, only two (Tan 2023 [46] and Virdee 2022 [49]) accounted for non-linearity of trends over time.

The analytical methods used to analyse blood test trends are reported in Table S7. Eight studies (28%) used only descriptive statistics/graphs [24,28,30,31,32,35,37,38]. Five studies (17%) used analytic methods designed for repeated measures data, including mixed-effects models, a generalised estimating equation, and joint modelling [21,29,33,40,49], whereas two (7%) used linear regression [34,41]. The analytic methods were unclear in one (3%) study. The remaining 13 (45%) used other methods, including logistic and Cox regression on the difference or percentage change between the two most recent tests, Cox regression with categorised trends, and t-tests comparing categorised trends between patients with and without cancer.

### 3.3. Cancers Associated with Blood Test Trends

**Figure 2:**
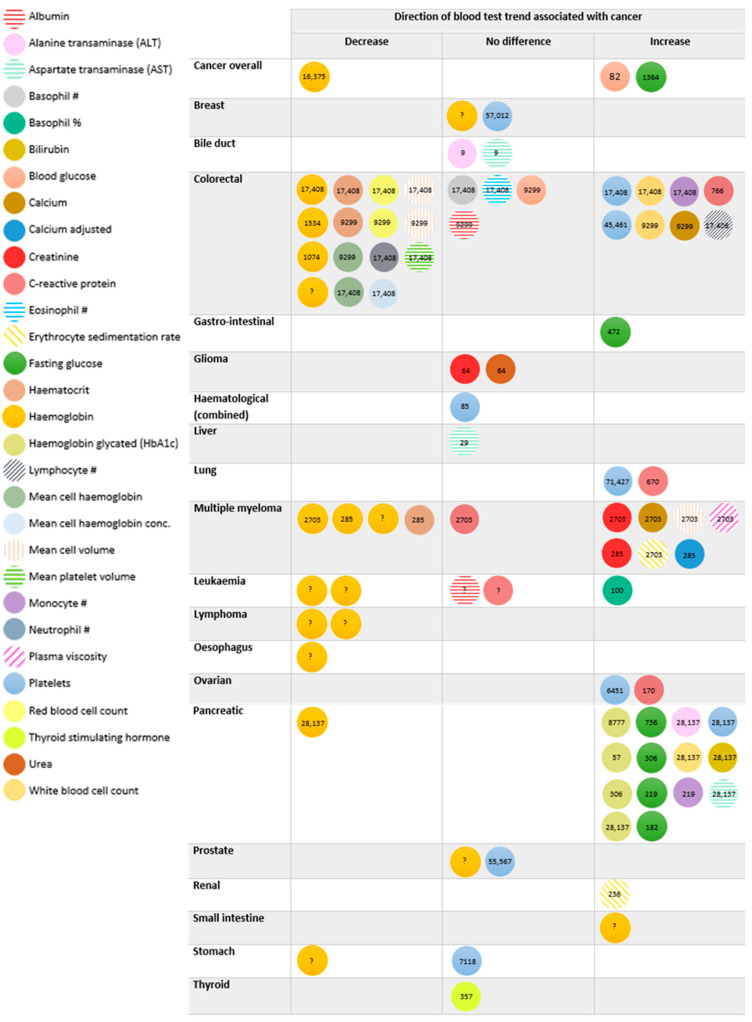
Overview of evidence of the association between blood test trends and undiagnosed cancer. Legend: This figure describes the direction of each blood test trend by cancer studied, and “no difference” indicates no difference in trend between patients with and without the cancer. The number in each circle is the number of cancer cases diagnosed in that study, and “?” indicates that the number of cancer cases was not reported in that study.

There were 25 cancers studied in total, with pancreatic (29%, n = 8) and colorectal (17%, n = 5) being the most commonly studied (Figure S2). Further details of cancer outcomes in each study are reported in Table S8. The direction of blood test trends associated with cancer are detailed in Figure 2, with further details in Tables S7 and S9. For example, increasing levels of HbA1c, platelets, ALT, AST, fasting glucose, white blood cell count, monocyte count, and calcium [24,33,35,39,41,43,44] and declining haemoglobin [46] were observed prior to pancreatic cancer diagnosis. A decline in red blood cell-related parameters [22,25,29,40,49] and a rise in white blood cell-related parameters, platelets, calcium, and C-reactive protein [22,47,49] was reported prior to colorectal cancer diagnosis, with no association between trends in albumin, blood glucose, basophil count, and eosinophil count [22,49] and colorectal cancer.

Of the 30 blood tests identified, an increasing pre-diagnostic trend in eight (27%) was associated with eight cancer types, and a decreasing pre-diagnostic trend in 17 (57%) was associated with 10 cancer types. For example, a declining haemoglobin level was observed prior to diagnosis of every cancer studied [21,25,29,37,40,46,49], except for breast and prostate cancer [25], where there was no association found, and small intestine cancer, where a rise was reported [25]. Additionally, a rising C-reactive protein was observed prior to diagnosis of colorectal, lung, and ovarian cancer [23,47,48], but no association was noted with multiple myeloma or leukaemia [30,37], and rising fasting glucose was noted prior to diagnosis of gastro-intestinal, pancreatic, and overall cancer [24,26,33,35,41].

No association was reported between trends in 11 (37%) tests, including albumin, urea, and thyroid stimulating hormone, and breast, bile duct, glioma, haematological combined, liver, prostate, or thyroid cancer [22,25,27,28,32,36,38,42]. Nine tests were not assessed in any study (Tables S4 and S5).

Examples of graphical trends published by studies can be found in Giannakeas 2022, Edgren 2010, Koshiaris 2018, and Virdee 2022 [25,28,37,49]. Graphical trends often showed a different trend in cases occurring years before diagnosis compared to controls, e.g., a rising fasting glucose for 3–4 years prior to diagnosis. The results of inferential/statistical analyses performed by each study, such as mixed-effects modelling, generalised estimating equations, and joint modelling, are shown in Table S7.

### 3.4. Cancer Staging and Blood Test Trend

One case–control study assessed trends in full blood count parameters prior to diagnosis of Duke’s A vs. D stage colorectal cancer [49]. No difference in graphical trends between Stage A and Stage D tumours were reported among older patients. However, changes in blood test results started up to one year earlier among Stage D compared to Stage A tumours in younger patients, observed in all FBC parameters except for mean platelet volume, basophil count, eosinophil count, and lymphocyte count for both males and females, where no difference between tumour stages was observed. The study used mixed-effects models to assess the association between tumour stage and blood test results over time. Compared to stage A tumours, stage D tumours were associated with increased platelets, white blood cell count, monocyte count, and neutrophil count for both males and females, lymphocyte count for females, and decreases in all red blood cell-related parameters, except red blood cell count and mean cell haemoglobin concentration, for males, where no different between tumour stage was reported. However, the study was limited to a small sample with available staging. No other study assessed the association between trends and tumour stage at diagnosis.

### 3.5. Blood Test Trend and Abnormality Thresholds

Single blood tests are considered abnormal in clinical practice if their values drop below or rise above a normal range, with some abnormalities known to be associated with increased risk of cancer. For example, the NICE guidelines suggest referral for colorectal cancer investigation if haemoglobin is less than 13 g/dL in men or 12 g/dL in women (anaemia, usually in the presence of other symptoms) [14]. Graphical trends in individual studies highlight that relevant differences in blood test trends between patients with and without cancer are, on average, confined within the normal range. For example, Edgren 2010 reported a declining haemoglobin, on average, confined within the normal range prior to diagnosis of oesophageal, stomach, colon, lymphoma, multiple myeloma, and lymphatic leukemia, with haemoglobin reaching the abnormal threshold/anaemia for myeloid/monocytic leukemia alone within approximately six months before diagnosis [25]. Additionally, Giannakeas 2022 reported a rising platelet count, on average, confined within the normal range (<400 × 10^9^/L—thrombocytosis) prior to diagnosis of colon, lung and stomach cancer [28]. Furthermore, Koshiaris 2018 reported an increasing calcium level, on average, prior to abnormality (>2.6 mmol/L) for multiple myeloma [37]. Graphical depictions suggest that blood test trend may pre-date blood test abnormality. However, no study compared the predictive ability (such as diagnostic accuracy) of blood test trend regarding blood test abnormality for cancer risk.

### 3.6. Risk of Bias

**Figure 3:**
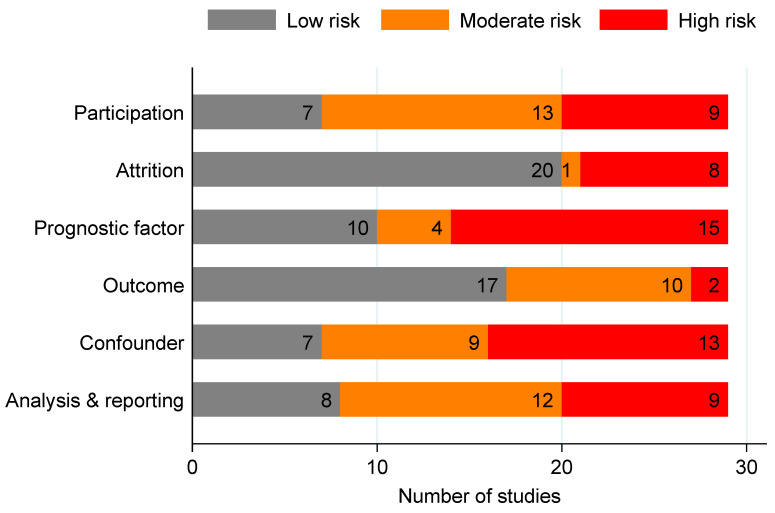
Summary of risk of bias scores, assessed using the QUIPS tool.

Risk of bias for each domain is summarised in Figure 3 and per study in Table S10. The studies were most commonly regarded as reflecting a high risk of bias for the prognostic factor domain (52%, n = 15), as the studies often did not use appropriate methods to handle missing data. The second-most frequent high risk of bias domain was the presence of confounders (45%, n = 13), as the studies often did not account for important confounders (age and sex, at minimum) in the study design or analysis. No study showed a high risk of bias in all domains. Atkin 2020 [21] scored the highest risk of bias, exhibiting a high risk in five of the six domains. Atkin 2020 did not adequately describe their patient eligibility criteria and study sample, clearly define prognostic variables and their outcome, adjust either their study design or analysis for important confounders, or present sufficient data to assess the adequacy of analysis.

## 4. Discussion

Our review has highlighted the blood test trends for which there is a reported association with undiagnosed cancer. We found evidence of an association between 25 blood test trends and 18 cancers. The most commonly reported blood test trends were a decreasing haemoglobin, associated with overall cancer, colorectal, multiple myeloma, leukaemia, lymphoma, oesophagus, pancreatic, and stomach cancer; a rising C-reactive protein, associated with a diagnosis of colorectal, lung and ovarian cancer; and an increasing fasting glucose, associated with overall cancer, gastro-intestinal, and pancreatic cancer.

No difference in pre-diagnostic blood test trends was reported by any study of breast, bile duct, glioma, haematological combined, liver, prostate, or thyroid cancer, although at most, two blood tests were assessed for each of these cancers. No study assessed the predictive ability of blood test trend when combined with symptoms, which may improve the identification of the cancer site and the choice of referral units, as a declining haemoglobin is associated with many cancer types. Some blood levels were not assessed by any study: red blood cell distribution width, white blood cell differential % (except basophil %), alkaline phosphatase, sodium, potassium, and amylase. Blood test trends were often not assessed for rare cancers. Some studies also used short periods of repeat blood testing, which may not be long enough to capture a predictive trend. Only one study assessed the association between blood test trend and cancer stage at diagnosis (for colorectal cancer), reporting associations between some full blood count parameters and Duke’s stage A vs. D, but this was limited by small sample sizes with available staging. No study compared the predictive ability of blood test trend over multiple tests to blood test abnormality on single tests, although graphical depictions of trends suggest they may identify cancer prior to blood test abnormality, facilitating earlier cancer detection.

### 4.1. Strengths and Limitations

To our knowledge, this is the first review regarding blood test trends for undiagnosed cancer. We performed a comprehensive search, developed with an information specialist, including full-length articles retrieved from MEDLINE and EMBASE. It is possible that additional relevant studies may be found exclusively in other databases and thus were missed by our review. However, it is likely that most relevant manuscripts were found, as MEDLINE and EMBASE provided a 97.5% coverage of articles in previous systematic reviews, and we conducted citation searching of all included manuscripts [50]. We were unable to apply our planned random-effects meta-analysis of effect estimates and were limited to a descriptive and narrative summary. This was because most blood tests and cancers were examined by too few studies and, while many studies assessed the same blood test for the same cancer, there was large variability in the methods used to assess the association between trend and cancer, so effect estimates were often not available.

### 4.2. Comparison with Existing Literature

Existing systematic reviews of cancer detection have focused on isolated tumour markers in relation to single cancers, such as serum HER2 for breast cancer [51]; IMP-1, CEA, and CA19-9 for pancreatic cancer [52]; and CEA, CA-125, PSA, and others for colorectal cancer [53]. We considered every cancer type and focused on tests that are commonly performed in clinical practice, as well as trends over repeatedly measured blood tests.

One recent review highlighted the diagnostic value of many full blood count parameters prior to colorectal cancer diagnosis and identified potential full blood count trends that could facilitate colorectal cancer detection [54]. Our review achieved similar findings, including a declining haemoglobin, mean cell volume, and red blood cell count and rising platelets and white blood cell count prior to colorectal cancer diagnosis. However, we also included other blood tests and cancers in our review.

Another recent clinical review focused on common blood tests as triage tests when used in isolation to provide “clues to cancer” in patients with non-specific symptoms attending primary care [14]. The review suggests that commonly performed blood tests should be considered in low-risk symptomatic patients. However, our review was not restricted to symptomatic patients and included all patient settings. We found relevant trends in these blood tests, with the potential to identify high risk patients. An increased cancer risk may be detected based on blood test trend prior to symptomatic presentation, but this possibility requires further investigation, as no studies included in our review reported the risk of cancer for symptoms and blood test trends combined.

### 4.3. Clinical and Research Implications

Current clinical guidelines do not include recommendations to investigate for cancer based on blood test trend. For example, current NICE guidance in the UK suggest referral for lung cancer investigation in adults aged 40+ years with thrombocytosis [15,55]. Clinicians consider blood test trends in practice based on plots or sequences of raw values. The role of blood test trends requires thorough investigation and testing in large real-world datasets. Giannakeas 2022 [28] reported that an increasing trend in platelets below the abnormal threshold (i.e., a trend confined within the normal range) is associated with lung cancer. NICE guidelines also suggest referral for investigation for colorectal, oesophageal, stomach, or other cancers in the presence of low haemoglobin [56]. We retrieved studies of haemoglobin, reporting that decreasing trends in haemoglobin within the normal range were associated with undiagnosed cancer [25,29,37,40,49]. Harnessing the full potential of blood test trends for cancer risk stratification may require the integration of more sophisticated trend analysis of electronic health records systems that are capable of analysing blood test trends in real-time. For this to occur, innovation is required in terms of both software and computational capacity. No study quantified the extent of additional benefits of blood test trends beyond decisions based on symptoms alone or symptoms plus single blood test abnormalities.

Sub-optimal methods to analyse trends were often identified. For example, most studies used inadequate methods, such as performing complete case analyses, to handle missing data. Methods to derive missing blood test data from other available blood test results have previously been reported (e.g., mean cell haemoglobin = haemoglobin/RBC) [57]. Some studies used Cox regression, incorporating blood test trend categories and linear regression, which is not appropriate for repeated measures data due to the fundamental assumption of the independence of data, with non-independent data known to bias confidence intervals and *p*-values. Recent methodological advancements, such as dynamic regression designed for repeated measures data, appropriately accounts for non-independent data and can handle unbalanced datasets, such as when blood test data are sporadically recorded in routine clinical practice [58]. Ongoing research aims to develop the evidence of the association between trends of blood tests used in clinical practice and cancer using large real-world datasets and to identify the most parsimonious methodology for longitudinal analysis to facilitate the integration of trends into risk stratification in clinical practice [59].

## 5. Conclusions

We highlight the cancers for which there is a reported association with changes in results over time in regards to blood tests commonly available in clinical practice, as well as the cancers and blood tests with no associated published literature. We also highlight further research areas that must be addressed in order to understand the value of blood test trends compared to the current standards. This review lays the foundation for further research and innovation in cancer detection.

## Figures and Tables

**Table 1:** Blood tests commonly available in clinical practice and included in this study.

Blood Test	Blood Level
Full Blood Count	red blood cell count, haemoglobin, haematocrit, mean cell volume, mean cell haemoglobin, mean cell haemoglobin concentration, red blood cell distribution width, platelet count, mean platelet volume, white blood cell count, basophil count, eosinophil count, lymphocyte count, monocyte count, neutrophil count, basophil %, eosinophil %, lymphocyte %, monocyte %, neutrophil %
Liver Function Tests	alanine aminotransaminase, albumin, alkaline phosphatase, aspartate transaminase, bilirubin
Renal Function	sodium, potassium, creatinine, urea
Inflammatory Markers	C-reactive protein, erythrocyte sedimentation rate, plasma viscosity
Other tests	amylase, HBA1c, calcium, calcium adjusted, total protein, blood glucose, fasting glucose, thyroid stimulating hormone

## Data Availability

Data are available from the authors, on request.

## Supplementary Materials

The following supporting information can be downloaded at: https://www.mdpi.com/article/10.3390/cancers16091692/s1, Table S1: Final search strategy (MEDLINE); Table S2: Final search strategy (EMBASE); Table S3: Detailed description of the 29 studies included in the review; Table S4: Full blood count trends analysed per study; Table S5: Liver function, renal, inflammatory, and other test trends analysed per study; Table S6: Blood test trends details and analytic approach per study; Table S7: Analytic methods and findings of the association between blood test trend and cancer; Table S8: Cancer outcome details per study; Table S9: Description of graphical blood test trend by cancer site; Table S10: Risk of bias in the 29 studies assessing the association between blood test trend and cancer, assessed using the QUIPS tool; Figure S1: Number of studies analysing each blood level; Figure S2: Number of studies studying each cancer site.
