# Supplementary material for: The Association between Blood Test Trends and Undiagnosed Cancer: A Systematic Review and Critical Appraisal
Source: Cancers (Basel). 2024 Apr 26;16(9):1692. doi: 10.3390/cancers16091692 (PMC11083147; doi:10.3390/cancers16091692)
Supplement: Supplementary file 1 [file cancers-16-01692-s001.zip › BLOTTED_SystematicReview_Supple_V2.0_05Mar2024.pdf]

Table S1: Final search strategy (MEDLINE)

MEDLINE
Database and platform: Medline (Ovid MEDLINE® Epub Ahead of Print, In-Process & Other Non-Indexed Citations, Ovid MEDLINE® Daily and Ovid MEDLINE®) 1946 to present. Search date: 15th May 2023.<br><br>1 exp Neoplasms/bl [Blood]<br>2 exp Neoplasms/<br>3 (neoplas* or tumor* or tumour* or cancer* or malignan* or carcino* or sarcom* or leukaem* or leukem* or lymphom* or melano* or metasta* or mesothelio* or mesotelio* or carcinomatos* or gliom* or glioblastom* or osteosarcom* or blastom* or neuroblastom* or oncolog* or myelodysplas* or adenocarcinoma* or choriocarcinoma*).ti.<br>4 2 or 3<br>5 Hematologic Tests/<br>6 exp Blood Cell Count/<br>7 exp Blood Cells/an<br>8 Blood Sedimentation/<br>9 Blood Viscosity/<br>10 exp Hemoglobins/an, bl<br>11 Hematocrit/<br>12 Erythrocyte Indices/ or Erythrocytes/an, bl<br>13 platelet function tests/ or mean platelet volume/<br>14 Liver Function Tests/<br>15 alanine transaminase/an, bl or exp aspartate aminotransferases/an, bl<br>16 Albumins/an or Albuminuria/bl, ur<br>17 serum albumin/ or serum albumin, human/<br>18 exp Bilirubin/an, bl<br>19 alpha-Fetoproteins/an, bl<br>20 Alkaline Phosphatase/an, bl [Analysis, Blood]<br>21 exp Kidney Function Tests/<br>22 Sodium/an, bl, ur<br>23 Potassium/an, bl, ur<br>24 Creatinine/an, bl, ur<br>25 Urea/an, bl, ur

26 Amylases/an, bl

27 Calcium/an, bl, ur

28 Glycated Hemoglobin A/an, bl or Blood Glucose/an, bl

29 Blood Proteins/an, bl

30 C-Reactive Protein/an, bl

31 exp Thyrotropin/an, bl

32 ((hemoglobin? or haemoglobin? or hb) adj3 (variation? or level? or concentration? or declin* or mean cell)).ti,ab,kf. or (hemoglobin? or haemoglobin?).ti.

33 ((complete blood or whole blood or blood cell or white cell or erythrocyte? or leukocyte? or platelet? or lymphocyte? or eosinophil? or neutrophil? or basophil? or monocyte?) adj2 (count? or variation?)).ti,ab,kf.

34 ((lymphocyte? or eosinophil? or neutrophil? or basophil? or monocyte?) adj2 (percent* or "%")).ti,ab,kf.

35 (erythrocyte sedimentation or blood sedimentation or blood viscosity or mean platelet or mean cell volume or hematocrit? or haematocrit? or ((blood cell or erythrocyte?) adj2 (index or indices or distribution))).ti,ab,kf.

36 ((c-reactive protein or crp) adj3 (plasma or serum or blood or variation? or level? or concentration? or elevat? or increas* or high*)).ti,ab,kf. or (c-reactive protein or crp).ti.

37 ((total or serum or blood or plasma) adj2 protein?).ti,ab,kf. or protein.ti.

38 ((albumin? adj3 (plasma or serum or blood or variation? or level? or concentration? or declin*)) or albumin creatinine ratio?).ti,ab,kf. or albumin*.ti.

39 ((alkaline phosphatase or alp) adj3 (plasma or serum or blood or variation? or level? or concentration? or elevat? or increas* or high*)).ti,ab,kf. or (alkaline phosphatase or alp).ti.

40 ((aminotransferase or ast or sgot or alt or sgpt) adj3 (plasma or serum or blood or variation? or level? or concentration? or elevat? or increas* or high*)).ti,ab,kf. or (aminotransferase or ast or sgot or alt or sgpt).ti.

41 (bilirubin adj3 (plasma or serum or blood or variation? or level? or concentration? or elevat? or increas* or high*)).ti,ab,kf. or bilirubin.ti.

42 (liver enzyme? adj3 (plasma or serum or blood or variation? or level? or concentration? or elevat? or increas* or high*)).ti,ab,kf. or (liver enzyme? or liver function).ti. or liver function test*.ti,ab,kf.

43 (renal funtion test* or kidney function test*).ti,ab,kf. or (renal function or kidney function).ti.

44 (sodium adj3 (plasma or serum or blood or variation? or level? or concentration? or declin*)).ti,ab,kf. or sodium.ti.

45 (potassium adj3 (plasma or serum or blood or variation? or level? or concentration? or elevat? or increas* or high*)).ti,ab,kf. or potassium.ti.

46 (creatinine adj3 (plasma or serum or blood or variation? or level? or concentration? or elevat? or increas* or high*)).ti,ab,kf. or creatinine.ti.

47 ((urea adj3 (plasma or serum or blood or variation? or level? or concentration? or decline?)) or urea cycle?).ti,ab,kf. or urea.ti.

48 (amylase adj3 (plasma or serum or blood or variation? or level? or concentration? or elevat? or increas* or high*)).ti,ab,kf. or amylase.ti.

49 (((glucose or hba1c) adj3 (plasma or serum or blood or variation? or level? or concentration? or elevat? or increas* or high*)) or fasting glucose).ti,ab,kf. or (glucose or hba1c).ti.

50 (calcium adj3 (plasma or serum or blood or variation? or level? or concentration? or elevat? or increas* or high*)).ti,ab,kf. or calcium.ti. or calcium adjusted.ti,ab,kf.

51 (((thyrotropin? or thyroid stimulating hormone?) adj3 (plasma or serum or blood or variation? or level? or concentration? or elevat? or increas* or high*)) or fasting glucose).ti,ab,kf. or (thyrotropon? or thyroid stimulating hormone?).ti.

52 anemia/ or anemia, hypochromic/ or anemia, iron-deficiency/ or exp anemia, macrocytic/

53 (an?emia? or an?emic or microcytosis or microcytic).ti,ab,kf.

54 or/5-53

55 4 and 54

56 1 or 55

57 exp Neoplasms/di

58 early diagnosis/ or "early detection of cancer"/

59 (detect* or diagnos* or screen*).ti.

60 ((neoplas* or tumor* or tumour* or cancer* or malignan* or carcino* or sarcom* or leukaem* or leukem* or lymphom* or melano* or metasta* or mesothelio* or mesotelio* or carcinomatos* or gliom* or glioblastom* or osteosarcom* or blastom* or neuroblastom* or oncolog* or myelodysplas* or adenocarcinoma* or choriocarcinoma*) adj3 (diagnos* or detect* or screen*)).ti,ab,kf.

61 57 or 58 or 59 or 60

62 56 and 61

63 (trend? or pattern? or lead time* or timeline* or time line* or time frame? or time frame? or interval?).ti,ab,kf.

64 ((longterm or long-term) adj2 (variation? or chang* or difference* or declin* or decreas* or increas* or elevat* or level? or concentration)).ti,ab,kf.

65 (prediagnos* or pre-diagnos* or ((before or prior) adj2 diagnos*)).ti,ab,kf.

66 ((risk? or predict*) adj5 (model* or logarithm* or algorithm* or maching learning)).ti,ab,kf.

67 (model* or logarithm* or algorithm* or maching learning).ti.

68 (risk? adj2 (scor* or model* or index or indices or tool* or assessment? or measurement?)).ti,ab,kf.

69 (Models, Biological/ or Models, Statistical/) and exp Risk/

70 63 or 64 or 65 or 66 or 67 or 68 or 69

71 62 and 70

72 exp animals/ not humans/

73 71 not 72

Table S2: Final search strategy (EMBASE)

EMBASE
Database and platform: Embase 1974 to present. Search date: 15th May 2023.<br><br>1 exp *neoplasm/<br>2 (neoplas* or tumor* or tumour* or cancer* or malignan* or carcino* or sarcom* or leukaem* or leukem* or lymphom* or melano* or metasta* or mesothelio* or mesotelio* or carcinomatos* or gliom* or glioblastom* or osteosarcom* or blastom* or neuroblastom* or oncolog* or myelodysplas* or adenocarcinoma* or choriocarcinoma*).ti.<br>3 1 or 2<br>4 hematological parameters/<br>5 exp blood cell count/<br>6 exp blood cell/an<br>7 erythrocyte sedimentation rate/<br>8 Blood Viscosity/<br>9 exp hemoglobin/an<br>10 hemoglobin blood level/ or "hemoglobin determination"/<br>11 Hematocrit/<br>12 exp erythrocyte parameters/<br>13 blood clotting parameters/ or exp platelet volume/<br>14 exp liver function test/<br>15 enzyme blood level/<br>16 alanine aminotransferase blood level/ or aminotransferase blood level/ or aspartate aminotransferase blood level/<br>17 protein blood level/<br>18 albumin blood level/<br>19 bilirubin blood level/<br>20 alpha fetoprotein blood level/<br>21 alkaline phosphatase blood level/<br>22 exp kidney function test/ or electrolyte blood level/<br>23 sodium blood level/<br>24 potassium blood level/<br>25 creatine kinase blood level/ or creatinine blood level/<br>26 urea blood level/

27 amylase blood level/

28 calcium blood level/

29 glucose blood level/ or exp *hemoglobin A1c/

30 C reactive protein/an

31 exp thyroid hormone blood level/ or thyrotropin blood level/

32 ((hemoglobin? or haemoglobin? or hb) adj3 (variation? or level? or concentration? or declin* or mean cell)).ti,ab,kf. or (hemoglobin? or haemoglobin?).ti.

33 ((complete blood or whole blood or blood cell or white cell or erythrocyte? or leukocyte? or platelet? or lymphocyte? or eosinophil? or neutrophil? or basophil? or monocyte?) adj2 (count? or variation?)).ti,ab,kf.

34 ((lymphocyte? or eosinophil? or neutrophil? or basophil? or monocyte?) adj2 (percent* or "%")).ti,ab,kf.

35 (erythrocyte sedimentation or blood sedimentation or blood viscosity or mean platelet or mean cell volume or hematocrit? or haematocrit? or ((blood cell or erythrocyte?) adj2 (index or indices or distribution))).ti,ab,kf.

36 ((c-reactive protein or crp) adj3 (plasma or serum or blood or variation? or level? or concentration? or elevat? or increas* or high*)).ti,ab,kf. or (c-reactive protein or crp).ti.

37 ((total or serum or blood or plasma) adj2 protein?).ti,ab,kf. or protein.ti.

38 ((albumin? adj3 (plasma or serum or blood or variation? or level? or concentration? or declin*)) or albumin creatinine ratio?).ti,ab,kf. or albumin*.ti.

39 ((alkaline phosphatase or alp) adj3 (plasma or serum or blood or variation? or level? or concentration? or elevat? or increas* or high*)).ti,ab,kf. or (alkaline phosphatase or alp).ti.

40 ((aminotransferase or ast or sgot or alt or sgpt) adj3 (plasma or serum or blood or variation? or level? or concentration? or elevat? or increas* or high*)).ti,ab,kf. or (aminotransferase or ast or sgot or alt or sgpt).ti.

41 (bilirubin adj3 (plasma or serum or blood or variation? or level? or concentration? or elevat? or increas* or high*)).ti,ab,kf. or bilirubin.ti.

42 (liver enzyme? adj3 (plasma or serum or blood or variation? or level? or concentration? or elevat? or increas* or high*)).ti,ab,kf. or (liver enzyme? or liver function).ti. or liver function test*.ti,ab,kf.

43 (renal funtion test* or kidney function test*).ti,ab,kf. or (renal function or kidney function).ti.

44 (sodium adj3 (plasma or serum or blood or variation? or level? or concentration? or declin*)).ti,ab,kf. or sodium.ti.

45 (potassium adj3 (plasma or serum or blood or variation? or level? or concentration? or elevat? or increas* or high*)).ti,ab,kf. or potassium.ti.

46 (creatinine adj3 (plasma or serum or blood or variation? or level? or concentration? or elevat? or increas* or high*)).ti,ab,kf. or creatinine.ti.

47 ((urea adj3 (plasma or serum or blood or variation? or level? or concentration? or decline?)) or urea cycle?).ti,ab,kf. or urea.ti.

48 (amylase adj3 (plasma or serum or blood or variation? or level? or concentration? or elevat? or increas* or high*)).ti,ab,kf. or amylase.ti.

49 (((glucose or hba1c) adj3 (plasma or serum or blood or variation? or level? or concentration? or elevat? or increas* or high*)) or fasting glucose).ti,ab,kf. or (glucose or hba1c).ti.

50 (calcium adj3 (plasma or serum or blood or variation? or level? or concentration? or elevat? or increas* or high*)).ti,ab,kf. or calcium.ti. or calcium adjusted.ti,ab,kf.

51 (((thyrotropin? or thyroid stimulating hormone?) adj3 (plasma or serum or blood or variation? or level? or concentration? or elevat? or increas* or high*)) or fasting glucose).ti,ab,kf. or (thyrotropon? or thyroid stimulating hormone?).ti.

52 anemia/ or exp iron deficiency anemia/ or exp macrocytic anemia/

53 (an?emia? or an?emic or microcytosis or microcytic).ti,ab,kf.

54 or/4-53

55 exp Neoplasm/di

56 early diagnosis/ or cancer diagnosis/ or early cancer diagnosis/

57 (detect* or diagnos* or screen*).ti.

58 ((neoplas* or tumor* or tumour* or cancer* or malignan* or carcino* or sarcom* or leukaem* or leukem* or lymphom* or melano* or metasta* or mesothelio* or mesotelio* or carcinomatos* or gliom* or glioblastom* or osteosarcom* or blastom* or neuroblastom* or oncolog* or myelodysplas* or adenocarcinoma* or choriocarcinoma*) adj3 (diagnos* or detect* or screen*)).ti,ab,kf.

59 55 or 56 or 57 or 58

60 (trend? or pattern? or lead time* or timeline* or time line* or time frame? or time frame? or interval?).ti,ab,kf.

61 ((longterm or long-term) adj2 (variation? or chang* or difference* or declin* or decreas* or increas* or elevat* or level? or concentration)).ti,ab,kf.

62 (prediagnos* or pre-diagnos* or ((before or prior) adj2 diagnos*)).ti,ab,kf.

63	((risk? or predict*) adj5 (model* or logarithm* or algorithm* or maching learning)).ti,ab,kf.
64	(model* or logarithm* or algorithm* or maching learning).ti.
65	(risk? adj2 (scor* or model* or index or indices or tool* or assessment? or measurement?)).ti,ab,kf.
66	(statistical model/ or biological model/ or disease model/ or cancer model/) and (risk/ or *risk assessment/)
67	60 or 61 or 62 or 63 or 64 or 65 or 66
68	3 and 54 and 59 and 67
69	exp animal/ not human/
70	68 not 69
71	conference*.pt.
72	70 not 71
73	70 and 71

Table S3: Detailed description of the 29 studies included in the review

Article	Study type	Study design	Geographic location	Patient setting	Reason for blood testing[1]	Patient population	Average age[2]	% female	No. blood tests	No. cancers
Atkin 2020	Retrospective	Case series	UK	Secondary care	Opportunistic tests	All patients diagnosed with multiple myeloma			4	1
Boursi 2016	Retrospective	Case-control	UK	Primary care	Opportunistic tests	All people receiving medical care from 1995 to 2013 from a THIN practitioner were eligible for inclusion. Subjects with a diagnosis of CRC syndromes, familial history of CRC, or IBD were excluded in order to focus on sporadic CRC. Patients without acceptable medical records were excluded (i.e., patients with incomplete documentation or out of sequence date of birth, registration date, date of death, or date of exit from the database).	69.72	52.7	8	1
Chaturvedi 2010	Retrospective	Case-control		Other	Screening for cancer	The PLCO study, a randomized trial aimed at evaluating the efficacy of screening in reducing cancer mortality, recruited approximately 155,000 men and women age 55 to 74 years from 1992 to 2001.	65-69	33.3	1	1
Edgren 2010	Retrospective	Case-control	Sweden and Denmark	Other	Opportunistic tests	We considered all donors for whom at least one successful whole blood, plasma or platelet donation was recorded between the January 1st, 1968 and December 31st, 2002, and who had no history of cancer at the time of the first recorded donation.	52.1	47	1	12

Feng 2020	Retrospective	Cohort	China	Unclear	Screening for cancer	Employees from the Kailuan Group older than 18 years including retired individuals from the Kailuan Group were invited to participate in health examinations. The current study was restricted to the population who participated in the examination in 2006 and at least one examination in 2008 and 2010. There were 101,139 individuals free from cancer who participated in the examination from 2006 to 2007. We excluded 1,222 participants without 2006 FBG data, 15,710 participants without FBG data or date of examination at 2008 and 2010, 12,845 participants with diabetes during 2006–2010 (self-reported history of diabetes, currently treated with insulin or oral hypoglycemic agents or a FBG concentration ≥7.0 mmol/l) and 1,620 participants who were either diagnosed with an incident cancer or died during the 2006–2010 period in which trajectories of FBG were assessed.	50	21.2	1	2
Fuente 2019	Retrospective	Case-control	USA	Other	Opportunistic tests	Only those who had either biopsy-proven PDAC or a pancreatic mass suspicious for adenocarcinoma with elevated CA19-9 or obstructive jaundice were included in the study as cases. For each confirmed PDAC case, we randomly selected 2 disease free age-(same birth year) and gender-matched Olmsted County residents as controls who were seen at the Mayo Clinic in the same calendar month as the matched cases date of PDAC diagnosis (index date).		49	1	1
Furukawa 1984	Unclear	Cohort	Japan	Unclear	Screening for cancer	114 patients with liver cirrhosis confirmed by laparoscopy and liver biopsy. None of them were treated with steroids.			1	1

Giannakeas 2022	Retrospective	Case series	Canada	Primary care	Opportunistic tests	Study subjects were patients with at least one complete blood count (CBC) record in the two-year period preceding or following a cancer diagnosis of the colon, lung, breast, prostate, stomach, or ovary.			1	6
Goldshtein 2010	Retrospective	Case-control	Israel	Primary care	Opportunistic tests	The study population included all MHS members aged 45–75 years who have been diagnosed with CRC between 1/1/2004 and 14/1/2009. We have excluded members whose Hb values were below normal ranges (11.7 g/dl for women and 12.6 g/dl for men) at any point during the first year of follow-up.			1	1
Gradel 2020	Retrospective	Case series	Denmark	Secondary care	Opportunistic tests	All patients with AML who were followed at the Department of Haematology, Odense University Hospital (OUH)	69.4	45.6	2	1
Hauser 2021	Retrospective	Cohort	USA	Primary care	Opportunistic tests	Patients with a BCR-ABL1 test during the study period. Patients prescribed a tyrosine kinase receptor inhibitor (eg, imatinib, dasatinib, nilotinib, bosutinib) at any time prior to their first BCR-ABL1 test were excluded. The patients in the study population were required to have at least 6 consecutive years of differential blood cell counts preceding the BCR-ABL1 test.	69	8	1	1
Hsieh 2019	Retrospective	Cohort	USA	Secondary care	Opportunistic tests	Adult patients (aged 18 years) seen at Mayo Clinic, Rochester, Minnesota, from January 1, 2011, through December 31, 2016. Patients who had at least 2 readings of platelet counts greater than 1000 109/L within 30 days of each other were identified.	57		1	1

						Patients who declined bone marrow studies despite physicians' recommendations were excluded (n¼3).				
Huang 2020	Retrospective	Cohort	USA	Unclear	Opportunistic tests	Patients were eligible for the study if they met the following inclusion criteria during 2006–2016: 45–90 years of age, 2 years of continuous membership, and available information for body mass index (BMI), smoking, and alcohol use. The 2-year membership requirement was used to ensure that individuals were in the KPSC system long enough to establish an accurate medical history. Otherwise eligible patients were further excluded if they did not have a glucose (fasting, random, or oral glucose tolerance) or HbA1c measurement (n ¼ 67,486), were not in the 4 major race or ethnicity groups (Asian, non-Hispanic black, Hispanic, non-Hispanic white) (n ¼ 80,801), or had a prior diagnosis of pancreatic cancer (n ¼ 523). Trends were assessed in the subgroup of diabetic patients only	57.9	55.2	2	1
Iversen 1996	Retrospective	Case-control	Norway	Unclear	Opportunistic tests	All cancer cases in Norway are reported to the Norwegien Cancer Registry. 500 RCC patients were selected. 130 were excluded as they had no pre-diagnostic ESR records. The results were compared to control data		44.7	1	1
Jacobson 2021	Prospective	Case-control	Sweden	Primary care	Screening for cancer (+ other conditions)	The NSHDS is a cohort study where the general population is invited to health examinations. We identified NSHDS participants that were subsequently diagnosed with pancreatic cancer in the	64.2	39	1	1

						Swedish cancer registry between January 1990 and December 2016. We used medical records to include only individuals with pancreatic ductal adenocarcinoma verified by histology or cytology. We excluded individuals with an uncertain diagnosis and those without records of fasting glucose, oral glucose tolerance tests (OGTT) or diabetes status. Controls without any cancer diagnosis were selected from the NSHDS.				
Jonsson 2020	Prospective	Case-control	Sweden	Primary care	Screening for cancer (+ other conditions)	Plasma samples were obtained from the Northern Sweden Health and Disease Study (NSHDS).Incident cases were diagnosed with glioma after the sampling. We identified 132 individuals diagnosed with glioma. We also randomly selected 132 controls (cancer-free at inclusion) with single or repeated samples. None of the cases had prior history of cancer.		78.1	2	1
Koshiaris 2018	Retrospective	Case-control	UK	Primary care	Opportunistic tests	Cases were selected if patients were >40 years of age with a myeloma diagnosis between January 2000 and December 2009. Date of diagnosis was defined as the first myeloma Read code, which was also used as the index date for the controls. Exclusion criteria included cases or controls with <1 year of records, cases without controls, controls with myeloma, and controls that did not seek medical care after registration.	73	47	7	1
Kubo 2016	Retrospective	Case series	Japan	Other	Screening for cancer	Nine patients with occupational cholangiocarcinoma (Table 1). Of the nine patients, six worked at a single printing company in Osaka	36.4	0	2	1

						(Company A) [2], two worked at another company (Company B), and one worked at third company (Company C) [4]. Of the nine patients, seven patients (patients 1, 3–8) were exposed to a high concentration of DCP, one (patient 9) was exposed to a high concentration of DCM and one (patient 2) was exposed to high concentrations of both DCP and DCM.				
Lemanska 2022	Retrospective	Case-control	UK	Primary care	Opportunistic tests	Practice inclusion was limited to practices passing data quality control. Participants were included in the matching process if they either had a pancreatic cancer diagnosis, or any of the listed above pancreatic cancer features at the age of 40+ years. The study sample included 3,539,397 adults registered within 590 practices.	72.1	49.8	1	1
Li 2021	Retrospective	Case-control	Sweden	Primary care	Opportunistic tests	All patients who had at least one hemoglobin test requested in primary care during 2000–2017, Aug 22 and who were above the age of 40 at the time of testing were identified. Colorectal cancer patients who have had no hemoglobin test in primary care prior to diagnosis, or only had post-CRC diagnostic hemoglobin tests in primary care were not eligible for the study.	70-79	49.4	1	1
Pannala 2009	Retrospective	Case-control	USA	Secondary care	Opportunistic tests	All outpatient FBG values in the Mayo records for 60 months before the index date for 1,172 cases and 2,344 controls. Of these individuals, the 736 cases and 1,875 controls who had at least one outpatient FBG value in the preceding 60 months or within one month following the index date (provided they received no	68.7	46.5	1	1

						treatment for pancreatic cancer) constituted the study population; further analyses were restricted to this subset of cases and controls.				
Rinaldi 2014	Prospective	Case-control	Europe	Primary care	Opportunistic tests	The EPIC cohort consists of approximately 370 000 women and 150 000 men apparently healthy and aged 35 to 69 years when recruited between 1992 and 1998 in 23 centers in 10 European countries. Participants were excluded if they reported a history of cancer other than nonmelanoma skin cancer. Thyroid cancer case patients with rare histological types (28 medullary, 6 anaplastic, and 3 other morphologies, and 1 lymphoma) were not included.		80	1	1
Sadr-Azodi 2015	Retrospective	Case-control	Sweden	Unclear	Opportunistic tests	All patients with diabetes mellitus in Sweden with a record in the Swedish National Diabetes Register (NDR) [14] and who had a dispensed prescription of anti-diabetic drugs. Individuals with missing information on the first recorded HbA 1c in the NDR (n=18 530), type of diabetes mellitus (n=23 280), duration of diabetes mellitus (n=35 248) and type of anti-diabetic treatment (n=3086) were excluded from the source population. In addition, patients with secondary diabetes mellitus (n=1515) and patients with a history of cancer diagnosis (excluding non-melanoma cancer of the skin) or cancer diagnosis (including neuroendocrine tumors of the pancreas) apart from pancreatic cancer during the follow-up (n=35 554) were also excluded.	67	44	1	1

Sharma 2018	Retrospective	Case-control	USA	Unclear	Opportunistic tests	All PDAC subjects in Olmsted County between 2000 and 2015, and manually reviewed their medical charts to include only those with a definite (confirmed by histopathology) or probable diagnosis of PDAC (pancreatic mass with elevated CA19–9 or obstructive jaundice). For each patient with PDAC we selected 2 age- (same birth year) and gender-matched Olmsted County residents as controls who were seen at the Mayo Clinic in the same calendar month as the matched patient's date of PDAC diagnosis (index date).	71.6	49	1	1
Stroud 2020	Prospective	Cohort	USA	Unclear	Screening for cancer	Participants aged 18+ years who underwent a primary bariatric surgery. All patients had no history of cancer, no cancer within 12 months post-operation, and at least 1 follow-up visit. Patients diagnosed without a diagnosis date or patients withdrawn between their 12-month post-operative and next scheduled visit were excluded	46	79.1	1	1
Tan 2023	Retrospective	Case-control	UK	Primary care	Opportunistic tests	Adult patients aged 18 years and above were eligible for inclusion and entered the study	72.08	50.3	7	1
Toriola 2011	Retrospective	Case-control	Finland	Other	Opportunistic tests	First trimester blood samples are withdrawn from pregnant women at the municipal maternity care units. ovarian cancer cases who had been pregnant on at least two occasions before cancer diagnosis and who had donated serum samples to the FMC during these different pregnancies were identified. Eligible		100	1	1

						controls were women from the FMC who were alive and free of cancer at the time of diagnosis of the case and had also donated at least two serum samples during different pregnancies.				
Toriola 2013	Retrospective	Case-control	USA	Other	Opportunistic tests	Women were eligible for the WHI-OS if they were postmenopausal, 50–79 years old and unlikely to relocate or die within 3 years.		100	1	1
Virdee 2022	Retrospective	Case-control	UK	Primary care	Opportunistic tests	Age 40+ years with at least one FBC blood test within 10 years before index date. Patients registered <1 year with their primary care practice, history of colorectal cancer before study entry, or diagnosed after study exit were excluded. Patients diagnosed with another cancer type before or simultaneously with colorectal cancer diagnosis were excluded. Patients with an available date of diagnosis but no indication of the cancer type were excluded.	60.32	57.5	14	1

[1]Opportunistic tests are those performed for various reasons but are being utilised for cancer assessment.
[2]Case-control studies: mean age at index date (diagnosis for cases, censor for controls). Other studies: mean age at study entry.

Table S4: Full blood count trends analysed per study

Article	RBC	Hb	Hc	MCV	MCH	MCHC	RDW	Plat	MPV	WBC	BasC	BasP	EosC	EosP	LymC	LymP	MonC	MonP	NeutC	NeutP
Atkin 2020		X	X																	
Boursi 2016	X		X	X	X					X										
Chaturvedi 2010																				
Edgren 2010		X																		
Feng 2020																				
Fuente 2019																	X			
Furukawa 1984																				
Giannakeas 2022								X												
Goldshtein 2010		X																		
Gradel 2020																				
Hauser 2021												X								
Hsieh 2019								X												
Huang 2020																				
Iversen 1996																				
Jacobson 2021																				
Jonsson 2020																				
Koshiaris 2018		X		X																
Kubo 2016																				
Lemanska 2022																				
Li 2021		X																		
Pannala 2009																				
Rinaldi 2014																				
Sadr-Azodi 2015																				
Sharma 2018																				
Stroud 2020																				
Tan 2023		X						X		X										
Toriola 2011																				
Toriola 2013																				

Virdee 2022	X	X	X	X	X	X		X	X	X	X		X		X		X		X	

Grey shaded columns indicate that the blood test was not assessed by any study.

Abbreviations: RBC=red blood cells, Hb=haemoglobin, Hc=haematocrit, MCV=mean corpuscular volume, MCH=mean corpuscular haemoglobin, MCHC=mean corpuscular haemoglobin concentration, RDW=red blood cell distribution width, Plat=platelet count, MPV=mean platelet volume, WBC=white blood cells, BasC=basophil count, BasP=basophil %, EosC=eosinophil count, EosP=eosinophil %, LymC=lymphocyte count, LymP=lymphocyte %, MonC=monocyte count, MonP=monocyte %, NeuC=neutrophil count, NeuP=neutrophil %

Table S5: Liver function, renal, inflammatory, and other test trends analysed per study

Article	Liver function test					Renal test				Inflammatory Markers			Other blood tests							
	ALT	Alb	AST	ALP	Bili	Sod	Pot	Creat	Urea	CRP	ESR	PV	Amy	HbGl	Calc	Calc adj	TP	BG	FG	TSH
Atkin 2020								X								X				
Boursi 2016		X													X			X		
Chaturvedi 2010										X										
Edgren 2010																				
Feng 2020																			X	
Fuente 2019																				
Furukawa 1984			X																	
Giannakeas 2022																				
Goldshtein 2010																				
Gradel 2020		X								X										
Hauser 2021																				
Hsieh 2019																				
Huang 2020														X					X	
Iversen 1996											X									
Jacobson 2021																			X	
Jonsson 2020								X	X											
Koshiaris 2018								X		X	X	X			X					
Kubo 2016	X		X																	
Lemanska 2022														X						
Li 2021																				
Pannala 2009																			X	
Rinaldi 2014																				X
Sadr-Azodi 2015														X						
Sharma 2018																			X	
Stroud 2020																		X		
Tan 2023	X		X		X									X						

Toriola 2011										X										
Toriola 2013										X										
Virdee 2022																				

Grey shaded columns indicate that the blood test was not assessed by any study.

Abbreviations: ALT=alanine aminotransaminase, Alb=albumin, AST=aspartate transaminase, Bili=bilirubin, Creat=creatinine, CRP=C-reactive protein, ESR=erythrocyte sedimentation rate, PV=plasma viscosity, Calc=calcium, Calc adj=calcium adjusted, BG=blood glucose, FG=fasting glucose, TSH= thyroid stimulating hormone

Figure S1: Number of studies analysing each blood level

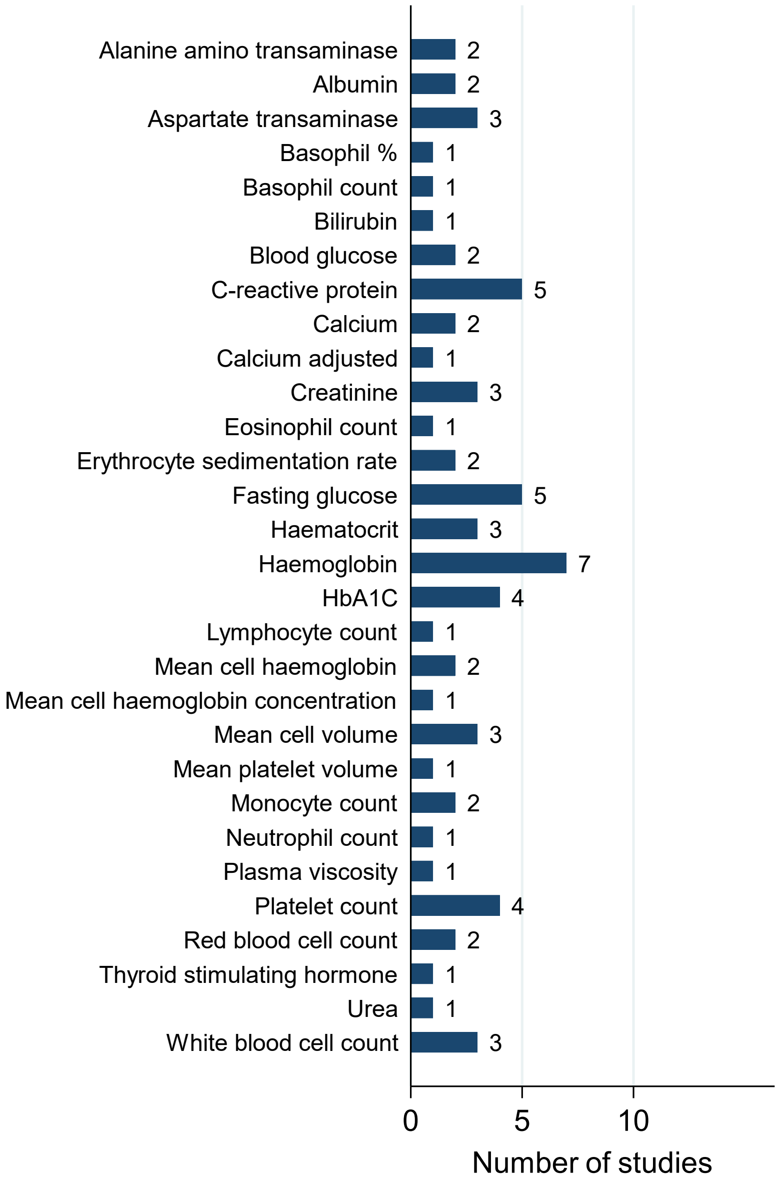

Table S6: Blood test trends details and analytic approach per study

Article	Analytic approach	Max longitudinal period for blood testing	Nature of repeat blood testing	If regularly taken, how often	Average no. blood tests used for trend	No. blood tests used in total
Atkin 2020	One-way repeated measures ANOVA	13 years	Sporadic			56711
Boursi 2016	Logistic regression on difference between two most recent tests	6.3 years	Sporadic		2	
Chaturvedi 2010	Logistic regression stratified by time between blood test and diagnosis	6 years	Regular intervals	1 year		
Edgren 2010	AUC stratified by time between blood test and diagnosis	5 years	Sporadic		10	
Edgren 2010	Descriptive/Graphs only	5 years	Sporadic		10	
Edgren 2010	Logistic regression stratified by time between blood test and diagnosis	5 years	Sporadic		10	
Feng 2020	Cox regression with trends categorised	4 years	Regular intervals	2 years	3	
Fuente 2019	Descriptive/Graphs only	2 years	Sporadic			
Furukawa 1984	T-test comparing groups derived based on trends patterns		Other			
Giannakeas 2022	Descriptive/Graphs only	2 years	Sporadic		2	
Goldshtein 2010	Mixed-effects models	10 years	Regular intervals	8 months		
Gradel 2020	Descriptive/Graphs only	30 days	Unclear			
Hauser 2021	Descriptive/Graphs only	6+ years	Sporadic			
Hsieh 2019	Descriptive/Graphs only	30 days	Unclear		2+	
Huang 2020	Generalised estimating equation	3 years	Unclear			
Iversen 1996	Linear regression including repeated blood tests	6 years	Sporadic			

Jacobson 2021	Descriptive/Graphs only		Unclear			
Jonsson 2020	Orthogonal projections to latent structures	11.7 years	Sporadic		2	256
Koshiaris 2018	Descriptive/Graphs only	5 years	Sporadic			
Kubo 2016	Descriptive/Graphs only	12 years	Regular intervals	1 year		
Lemanska 2022	Logistic regression per time interval between blood test and diagnosis	6 years	Sporadic			
Li 2021	Mixed-effects models	16.5 years	Sporadic		2	9486
Pannala 2009	Linear regression for cases and controls separately	5 years	Sporadic			
Rinaldi 2014	Unclear	>11 years	Unclear			
Sadr-Azodi 2015	Logistic regression per time interval between blood test and diagnosis	>5 years	Sporadic			
Sharma 2018	Linear regression per time interval between blood test and diagnosis	5 years	Sporadic		3.5	
Sharma 2018	T-test stratified by time	5 years	Sporadic		3.5	
Stroud 2020	Cox regression on percentage change	12 months	Regular intervals	1 year	2	
Tan 2023	Joinpoint regression for cases and controls separately	5 years	Sporadic			
Toriola 2011	Logistic regression on percentage change	15 years	Other		2	
Toriola 2011	Logistic regression per time interval between blood test and diagnosis	15 years	Other		2	
Toriola 2013	T-test of percentage change	3 years	Other		2	
Virdee 2022	Univariate joint models	10 years	Sporadic			3068228

Figure S2: Number of studies studying each cancer site

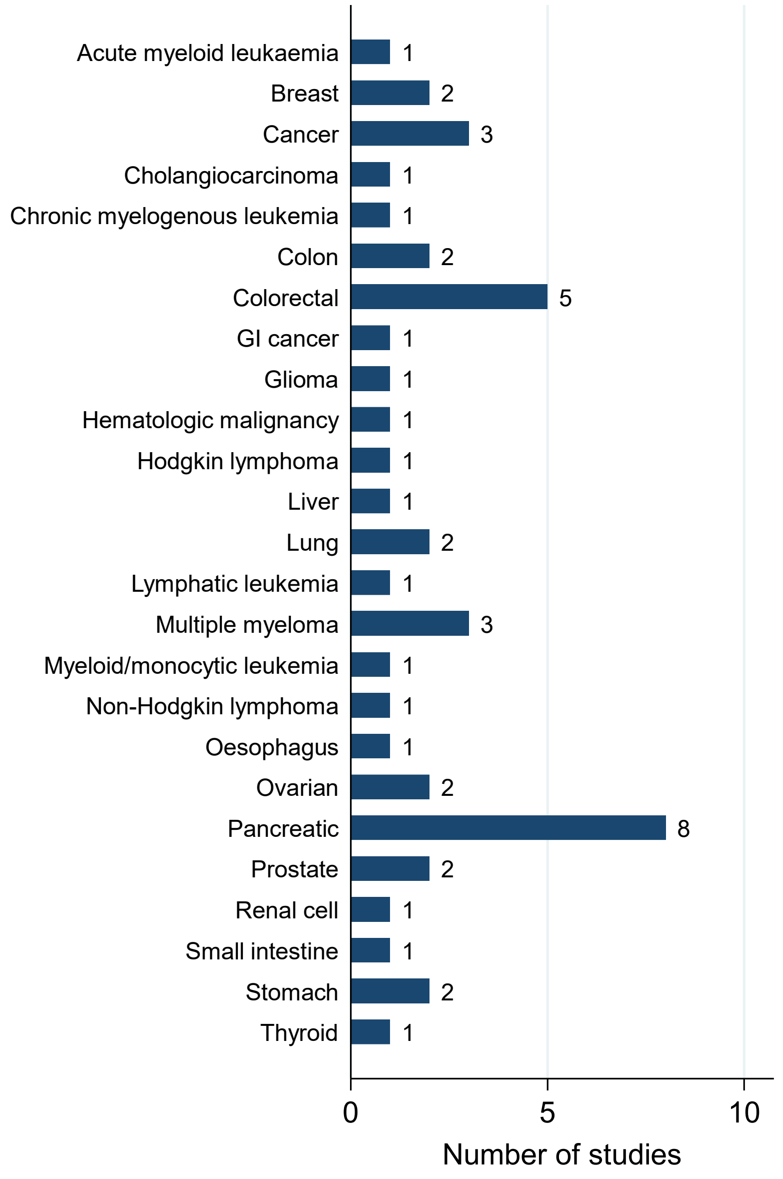

Table S7: Analytic methods and findings of the association between blood test trend and cancer

Cancer	Blood Level	Risk window[1]	Strata (if applicable)	Analytic approach	Results	Article
Breast	Haemoglobin	-1 year		Logistic regression stratified by time	OR=1.00 (0.95–1.05)	Edgren 2010
Breast	Haemoglobin	-1-2 years		AUC stratified by time	AUC=0.51 (0.49-0.52)	Edgren 2010
Breast	Haemoglobin	-1-2 years		Logistic regression stratified by time	OR=0.98 (0.93–1.03)	Edgren 2010
Breast	Haemoglobin	-2-3 years		Logistic regression stratified by time	OR=1.00 (0.95–1.06)	Edgren 2010
Breast	Haemoglobin	-3-4 years		Logistic regression stratified by time	OR=0.93 (0.88–0.99)	Edgren 2010
Breast	Haemoglobin	-4-5 years		Logistic regression stratified by time	OR=0.99 (0.93–1.05)	Edgren 2010
Cancer	Blood glucose	7 years		Cox regression on percentage change	n=1572, cases=62, HR=%change=0.94 (0.90-0.99). Multivariable, adjusted for: Age at surgery, education, smoking history, insulin use, 12-month percentage weight change	Stroud 2020
Cancer	Fasting glucose	6 years		Cox regression with trends categorised	Group 1=Low-increasing, n=6275, cases=152, HR=1.26 (1.06–1.50). Group 2 (Ref)=Moderate-stable, n=44120, cases=818. Group 3=Moderate-increasing, n=10149, cases=215, HR=1.05 (0.90–1.22). Group 4=Elevated-decreasing, n=5244, cases=87, HR=0.93 (0.74–1.15). Group 5=Elevated-stable, n=3954, cases=92, HR=1.13 (0.91–1.41). Multivariable, adjusted for: Age, sex, income,	Feng 2020

					educational level, smoking, alcohol drinking, BMI at 2006, BMI percent change during 2006–2010	
Cancer	Haemoglobin	-1 year		Logistic regression stratified by time	OR=1.06 (1.04–1.09)	Edgren 2010
Cancer	Haemoglobin	-1-2 years		AUC stratified by time	AUC=0.53 (0.53-0.54)	Edgren 2010
Cancer	Haemoglobin	-1-2 years		Logistic regression stratified by time	OR=0.99 (0.97–1.02)	Edgren 2010
Cancer	Haemoglobin	-2-3 years		Logistic regression stratified by time	OR=0.99 (0.96–1.01)	Edgren 2010
Cancer	Haemoglobin	-3-4 years		Logistic regression stratified by time	OR=0.95 (0.93–0.97)	Edgren 2010
Cancer	Haemoglobin	-4-5 years		Logistic regression stratified by time	OR=0.96 (0.94–0.99)	Edgren 2010
Colon	Haemoglobin	-1 year		Logistic regression stratified by time	OR=1.82 (1.64–2.01)	Edgren 2010
Colon	Haemoglobin	-1-2 years		AUC stratified by time	AUC=0.62 (0.59-0.64)	Edgren 2010
Colon	Haemoglobin	-1-2 years		Logistic regression stratified by time	OR=1.19 (1.08–1.32)	Edgren 2010
Colon	Haemoglobin	-2-3 years		Logistic regression stratified by time	OR=1.06 (0.96–1.17)	Edgren 2010
Colon	Haemoglobin	-3-4 years		Logistic regression stratified by time	OR=1.07 (0.97–1.18)	Edgren 2010

Colon	Haemoglobin	-4-5 years		Logistic regression stratified by time	OR=0.96 (0.87–1.05)	Edgren 2010
Colorectal	Albumin	Unclear		Logistic regression on difference between two most recent tests	n=9299, cases=36199, OR=0.84 (0.70-1.02)	Boursi 2016
Colorectal	Basophil count	Diagnosis	Females	Univariate joint models	n=50000, cases=788, OR=Decline=0.225 (0.028, 1.835). Multivariable, adjusted for: Age at index (diagnosis, random)	Virdee 2022
Colorectal	Basophil count	Diagnosis	Males	Univariate joint models	n=50000, cases=1207, OR=Decline=0.210 (0.040, 1.101). Multivariable, adjusted for: Age at index (diagnosis, random)	Virdee 2022
Colorectal	Blood glucose	Unclear		Logistic regression on difference between two most recent tests	n=9299, cases=36199, OR=0.96 (0.91-1.01)	Boursi 2016
Colorectal	C-reactive protein	Unclear		T-test of percentage change	Group 1=Case baseline (n=766), Group 2=Case 3 years (n=766), Results=0.58 (0.01 – 1.14)	Toriola 2013
Colorectal	C-reactive protein	Unclear		T-test of percentage change	Group 1=Control baseline (n=875), Group 2=Control 3 years (n=875), Results=controls: -0.04 (-0.56 – 0.47)	Toriola 2013
Colorectal	Calcium	Unclear		Logistic regression on difference between two most recent tests	n=9299, cases=36199, OR=1.9 (0.65-5.52)	Boursi 2016
Colorectal	Eosinophil count	Diagnosis	Females	Univariate joint models	n=50000, cases=796, HR=Decline=0.484 (0.252, 0.929). Multivariable, adjusted for: Age at index (diagnosis, random)	Virdee 2022
Colorectal	Eosinophil count	Diagnosis	Males	Univariate joint models	n=50000, cases=1233, HR=Decline=0.689 (0.440, 1.079). Multivariable, adjusted for: Age at index (diagnosis, random)	Virdee 2022

Colorectal	Haematocrit/Packed cell volume	Diagnosis	Females	Univariate joint models	n=50000, cases=775, HR=Decline=1.001 (1.001, 1.001). Multivariable, adjusted for: Age at index (diagnosis, random)	Virdee 2022
Colorectal	Haematocrit/Packed cell volume	Diagnosis	Males	Univariate joint models	n=50000, cases=1274, HR=Decline=1.001 (1.001, 1.001). Multivariable, adjusted for: Age at index (diagnosis, random)	Virdee 2022
Colorectal	Haematocrit/Packed cell volume	Unclear		Logistic regression on difference between two most recent tests	n=9299, cases=36199, OR=1.04 (0.98-1.11)	Boursi 2016
Colorectal	Haemoglobin	Diagnosis		Mixed-effects models	Method: Mixed-effects models. Result: Male controls: Hb=0.09ln(time)+14.49, R2=0.83), male cases: Hb=0.48ln(time)+14.12, R2=0.98); female controls: Hb=0.04ln(time)+13.111, R2=0.65), female cases: Hb=0.3ln(time)+12.72, R2=0.97)	Goldshtein 2010
Colorectal	Haemoglobin	Diagnosis		Mixed-effects models	Method: Mixed-effects models. Result: No results reported	Li 2021
Colorectal	Haemoglobin	Diagnosis	Females	Univariate joint models	n=50000, cases=747, HR=Decline=2.037 (1.953, 2.128). Multivariable, adjusted for: Age at index (diagnosis, random)	Virdee 2022
Colorectal	Haemoglobin	Diagnosis	Males	Univariate joint models	n=50000, cases=1230, HR=Decline=1.783 (1.730, 1.835). Multivariable, adjusted for: Age at index (diagnosis, random)	Virdee 2022
Colorectal	Lymphocyte count	Diagnosis	Females	Univariate joint models	n=50000, cases=775, HR=Decline=1.215 (1.098, 1.346). Multivariable, adjusted for: Age at index (diagnosis, random)	Virdee 2022
Colorectal	Lymphocyte count	Diagnosis	Males	Univariate joint models	n=50000, cases=1222, HR=Decline=1.235 (1.139, 1.339). Multivariable, adjusted for: Age at index (diagnosis, random)	Virdee 2022

Colorectal	Mean cell haemoglobin	Diagnosis	Females	Univariate joint models	n=50000, cases=716, HR=Decline=1.333 (1.292, 1.376). Multivariable, adjusted for: Age at index (diagnosis, random)	Virdee 2022
Colorectal	Mean cell haemoglobin	Diagnosis	Males	Univariate joint models	n=50000, cases=1134, HR=Decline=1.376 (1.342, 1.410). Multivariable, adjusted for: Age at index (diagnosis, random)	Virdee 2022
Colorectal	Mean cell haemoglobin	Unclear		Logistic regression on difference between two most recent tests	n=9299, cases=36199, OR=1.27 (1.04-1.55)	Boursi 2016
Colorectal	Mean cell haemoglobin concentration	Diagnosis	Females	Univariate joint models	n=50000, cases=736, HR=Decline=1.862 (1.748, 1.984). Multivariable, adjusted for: Age at index (diagnosis, random)	Virdee 2022
Colorectal	Mean cell haemoglobin concentration	Diagnosis	Males	Univariate joint models	n=50000, cases=1178, HR=Decline=2.169 (2.053, 2.288). Multivariable, adjusted for: Age at index (diagnosis, random)	Virdee 2022
Colorectal	Mean cell volume	Diagnosis	Females	Univariate joint models	n=50000, cases=758, HR=Decline=1.110 (1.100, 1.121). Multivariable, adjusted for: Age at index (diagnosis, random)	Virdee 2022
Colorectal	Mean cell volume	Diagnosis	Males	Univariate joint models	n=50000, cases=1181, HR=Decline=1.124 (1.116, 1.133). Multivariable, adjusted for: Age at index (diagnosis, random)	Virdee 2022
Colorectal	Mean cell volume	Unclear		Logistic regression on difference between two most recent tests	n=9299, cases=36199, OR=1.23 (1.01-1.51)	Boursi 2016
Colorectal	Mean platelet volume	Diagnosis	Females	Univariate joint models	n=50000, cases=734, HR=Decline=1.155 (1.092, 1.221). Multivariable, adjusted for: Age at index (diagnosis, random)	Virdee 2022

Colorectal	Mean platelet volume	Diagnosis	Males	Univariate joint models	n=50000, cases=1901, HR=Decline=1.142 (1.089, 1.196). Multivariable, adjusted for: Age at index (diagnosis, random)	Virdee 2022
Colorectal	Monocyte count	Diagnosis	Females	Univariate joint models	n=50000, cases=784, HR=Increase=2.210 (1.691, 2.889). Multivariable, adjusted for: Age at index (diagnosis, random)	Virdee 2022
Colorectal	Monocyte count	Diagnosis	Males	Univariate joint models	n=50000, cases=1164, HR=Increase=1.729 (1.463, 2.043). Multivariable, adjusted for: Age at index (diagnosis, random)	Virdee 2022
Colorectal	Neutrophil count	Diagnosis	Females	Univariate joint models	n=50000, cases=785, HR=Decline=1.093 (1.065, 1.121). Multivariable, adjusted for: Age at index (diagnosis, random)	Virdee 2022
Colorectal	Neutrophil count	Diagnosis	Males	Univariate joint models	n=50000, cases=1186, HR=Decline=1.018 (0.970, 1.068). Multivariable, adjusted for: Age at index (diagnosis, random)	Virdee 2022
Colorectal	Platelet count	Diagnosis	Females	Univariate joint models	n=50000, cases=718, HR=Increase=1.007 (1.006, 1.007). Multivariable, adjusted for: Age at index (diagnosis, random)	Virdee 2022
Colorectal	Platelet count	Diagnosis	Males	Univariate joint models	n=50000, cases=1107, HR=Increase=1.007 (1.006, 1.007). Multivariable, adjusted for: Age at index (diagnosis, random)	Virdee 2022
Colorectal	Red blood cell count	Diagnosis	Females	Univariate joint models	n=50000, cases=734, HR=Decline=2.551 (2.132, 3.049). Multivariable, adjusted for: Age at index (diagnosis, random)	Virdee 2022
Colorectal	Red blood cell count	Diagnosis	Males	Univariate joint models	n=50000, cases=1135, HR=Decline=1.957 (1.706, 2.242). Multivariable, adjusted for: Age at index (diagnosis, random)	Virdee 2022

Colorectal	Red blood cell count	Unclear		Logistic regression on difference between two most recent tests	n=9299, cases=36199, OR=1.09 (0.97-1.23)	Boursi 2016
Colorectal	White blood cell count	Diagnosis	Females	Univariate joint models	n=50000, cases=809, HR=Increase=1.047 (0.973, 1.128). Multivariable, adjusted for: Age at index (diagnosis, random)	Virdee 2022
Colorectal	White blood cell count	Diagnosis	Males	Univariate joint models	n=50000, cases=1203, HR=Increase=1.054 (1.012, 1.098). Multivariable, adjusted for: Age at index (diagnosis, random)	Virdee 2022
Colorectal	White blood cell count	Unclear		Logistic regression on difference between two most recent tests	n=9299, cases=36199, OR=0.97 (0.94-1.00)	Boursi 2016
GI cancer	Fasting glucose	6 years		Cox regression with trends categorised	Group 1=Low-increasing, n=6275, cases=39, OR=0.97 (0.69–1.36). Group 2 (Ref)=Moderate-stable, n=44120, cases=282. Group 3=Moderate-increasing, n=10149, cases=71, OR=0.97 (0.75–1.26). Group 4=Elevated-decreasing, n=5244, cases=31, OR=0.91 (0.63–1.32). Group 5=Elevated-stable, n=3954, cases=49, OR=1.66 (1.22–2.26). Multivariable, adjusted for: Age, sex, income, educational level, smoking, alcohol drinking, BMI at 2006, BMI percent change during 2006–2010	Feng 2020
Glioma	Creatinine	Diagnosis		Orthogonal projections to latent structures	Method: Orthogonal projections to latent structures. Result: No results reported	Jonsson 2020
Glioma	Urea	Diagnosis		Orthogonal projections to latent structures	Method: Orthogonal projections to latent structures. Result: No results reported	Jonsson 2020
Hodgkin lymphoma	Haemoglobin	-1 year		Logistic regression stratified by time	OR=1.72 (1.37–2.16)	Edgren 2010

Hodgkin lymphoma	Haemoglobin	-1-2 years		AUC stratified by time	AUC=0.59 (0.53-0.56)	Edgren 2010
Hodgkin lymphoma	Haemoglobin	-1-2 years		Logistic regression stratified by time	OR=1.26 (1.00–1.59)	Edgren 2010
Hodgkin lymphoma	Haemoglobin	-2-3 years		Logistic regression stratified by time	OR=0.99 (0.80–1.24)	Edgren 2010
Hodgkin lymphoma	Haemoglobin	-3-4 years		Logistic regression stratified by time	OR=0.98 (0.78–1.23)	Edgren 2010
Hodgkin lymphoma	Haemoglobin	-4-5 years		Logistic regression stratified by time	OR=0.87 (0.69–1.11)	Edgren 2010
Liver	Aspartate transaminase	Diagnosis		T-test comparing groups derived based on trends patterns	Group 1=fluctuating trend over >20ng/mL (n=18), Group 2=Type 3: <20ng/ml throughout (n=8), Results=p<0.01	Furukawa 1984
Liver	Aspartate transaminase	Diagnosis		T-test comparing groups derived based on trends patterns	Group 1=fluctuating trend over >20ng/mL (n=18), Group 2=spike shaped rise but mostly <20ng/ml (n=3), Results=p<0.05	Furukawa 1984
Lung	C-reactive protein	1 year		Logistic regression stratified by time	Group 1 (Ref)=<=1; n=274, cases=168. Group 2=1.1-2.7; n=312, cases=176. Group 3=2.8-5.5; n=317, cases=167. Group 4=>=5.6; n=359, cases=159. All HRs statistically significant	Chaturvedi 2010
Lung	C-reactive protein	1-2 years		Logistic regression stratified by time	Group 1 (Ref)=<=1. Group 2=1.1-2.7. Group 3=2.8-5.5. Group 4=>=5.6. All HRs statistically significant	Chaturvedi 2010
Lung	C-reactive protein	2-5 years		Logistic regression stratified by time	Group 1 (Ref)=<=1. Group 2=1.1-2.7. Group 3=2.8-5.5. Group 4=>=5.6. All HRs statistically significant	Chaturvedi 2010
Lung	C-reactive protein	5+ years		Logistic regression stratified by time	Group 1 (Ref)=<=1. Group 2=1.1-2.7. Group 3=2.8-5.5. Group 4=>=5.6. All HRs statistically significant	Chaturvedi 2010

Lung	C-reactive protein	Diagnosis		Logistic regression stratified by time	Group 1 (Ref)=<=1. Group 2=1.1-2.7. Group 3=2.8-5.5. Group 4=>=5.6. All HRs statistically significant	Chaturvedi 2010
Lymphatic leukemia	Haemoglobin	-1 year		Logistic regression stratified by time	OR=1.74 (1.42–2.13)	Edgren 2010
Lymphatic leukemia	Haemoglobin	-1-2 years		AUC stratified by time	AUC=0.55 (0.49-0.60)	Edgren 2010
Lymphatic leukemia	Haemoglobin	-1-2 years		Logistic regression stratified by time	OR=1.35 (1.11–1.65)	Edgren 2010
Lymphatic leukemia	Haemoglobin	-2-3 years		Logistic regression stratified by time	OR=1.38 (1.12–1.70)	Edgren 2010
Lymphatic leukemia	Haemoglobin	-3-4 years		Logistic regression stratified by time	OR=1.08 (0.89–1.31)	Edgren 2010
Lymphatic leukemia	Haemoglobin	-4-5 years		Logistic regression stratified by time	OR=1.28 (1.05–1.56)	Edgren 2010
Multiple myeloma	Calcium adjusted	Diagnosis		One-way repeated measures ANOVA	Group 1=0-90 days, Group 2=90-180 days, Group 3=180-210 days. P=Unknown	Atkin 2020
Multiple myeloma	Creatinine	Diagnosis		One-way repeated measures ANOVA	Group 1=0-90 days, Group 2=90-180 days, Group 3=180-210 days. P=Unknown	Atkin 2020
Multiple myeloma	Haematocrit/Packed cell volume	Diagnosis		One-way repeated measures ANOVA	Group 1=0-90 days, Group 2=90-180 days, Group 3=180-210 days. P=Not significant	Atkin 2020
Multiple myeloma	Haemoglobin	-1 year		Logistic regression stratified by time	OR=2.50 (1.94–3.23)	Edgren 2010
Multiple myeloma	Haemoglobin	-1-2 years		AUC stratified by time	AUC=0.71 (0.65-0.78)	Edgren 2010

Multiple myeloma	Haemoglobin	-1-2 years		Logistic regression stratified by time	OR=1.58 (1.24–2.02)	Edgren 2010
Multiple myeloma	Haemoglobin	-2-3 years		Logistic regression stratified by time	OR=1.42 (1.14–1.77)	Edgren 2010
Multiple myeloma	Haemoglobin	-3-4 years		Logistic regression stratified by time	OR=1.21 (0.96–1.52)	Edgren 2010
Multiple myeloma	Haemoglobin	-4-5 years		Logistic regression stratified by time	OR=1.13 (0.90–1.43)	Edgren 2010
Multiple myeloma	Haemoglobin	Diagnosis		One-way repeated measures ANOVA	Group 1=0-90 days, Group 2=90-180 days, Group 3=180-210 days. P=.0005	Atkin 2020
Myeloid/monocytic leukemia	Haemoglobin	-1 year		Logistic regression stratified by time	OR=2.29 (1.82–2.89)	Edgren 2010
Myeloid/monocytic leukemia	Haemoglobin	-1-2 years		AUC stratified by time	AUC=0.71 (0.65-0.77)	Edgren 2010
Myeloid/monocytic leukemia	Haemoglobin	-1-2 years		Logistic regression stratified by time	OR=1.14 (0.94–1.39)	Edgren 2010
Myeloid/monocytic leukemia	Haemoglobin	-2-3 years		Logistic regression stratified by time	OR=1.08 (0.87–1.35)	Edgren 2010
Myeloid/monocytic leukemia	Haemoglobin	-3-4 years		Logistic regression stratified by time	OR=1.14 (0.92–1.40)	Edgren 2010
Myeloid/monocytic leukemia	Haemoglobin	-4-5 years		Logistic regression stratified by time	OR=1.01 (0.82–1.24)	Edgren 2010
Non-Hodgkin lymphoma	Haemoglobin	-1 year		Logistic regression stratified by time	OR=1.30 (1.15–1.48)	Edgren 2010

Non-Hodgkin lymphoma	Haemoglobin	-1-2 years		AUC stratified by time	AUC=0.57 (0.54-0.60)	Edgren 2010
Non-Hodgkin lymphoma	Haemoglobin	-1-2 years		Logistic regression stratified by time	OR=1.06 (0.94–1.19)	Edgren 2010
Non-Hodgkin lymphoma	Haemoglobin	-2-3 years		Logistic regression stratified by time	OR=0.98 (0.87–1.10)	Edgren 2010
Non-Hodgkin lymphoma	Haemoglobin	-3-4 years		Logistic regression stratified by time	OR=0.91 (0.81–1.03)	Edgren 2010
Non-Hodgkin lymphoma	Haemoglobin	-4-5 years		Logistic regression stratified by time	OR=0.95 (0.85–1.07)	Edgren 2010
Oesophagus	Haemoglobin	-1 year		Logistic regression stratified by time	OR=1.22 (0.94–1.57)	Edgren 2010
Oesophagus	Haemoglobin	-1-2 years		AUC stratified by time	AUC=0.60 (0.51-0.69)	Edgren 2010
Oesophagus	Haemoglobin	-1-2 years		Logistic regression stratified by time	OR=1.12 (0.85–1.49)	Edgren 2010
Oesophagus	Haemoglobin	-2-3 years		Logistic regression stratified by time	OR=0.98 (0.74–1.30)	Edgren 2010
Oesophagus	Haemoglobin	-3-4 years		Logistic regression stratified by time	OR=1.01 (0.77–1.34)	Edgren 2010
Oesophagus	Haemoglobin	-4-5 years		Logistic regression stratified by time	OR=1.01 (0.77–1.34)	Edgren 2010
Ovarian	C-reactive protein	Diagnosis		Logistic regression on percentage change	Group 1 (Ref)=Little change; n=206, cases=91. Group 2=Substantial decrease; n=33, cases=17, OR=1.56 (0.71–3.45). Group 3=Substantial increase; n=101, cases=62, OR=1.90 (1.12–3.23)	Toriola 2011

Ovarian	C-reactive protein	Diagnosis	Sample 1	Logistic regression per time-point	Group 1 (Ref)=<1.1; n=104, cases=47. Group 2=1.1 to <=2.6; n=113, cases=56, OR=1.35 (0.77–2.35). Group 3=>2.6; n=123, cases=67, OR=1.62 (0.93–2.83). Multivariable, adjusted for: Age at blood test	Toriola 2011
Ovarian	C-reactive protein	Diagnosis	Sample 2	Logistic regression per time-point	Group 1 (Ref)=<1.1; n=104, cases=48. Group 2=1.1 to <=2.6; n=104, cases=44, OR=0.97 (0.56–1.62). Group 3=>2.6; n=132, cases=78, OR=1.96 (1.11–3.49). Multivariable, adjusted for: Age at blood test	Toriola 2011
Pancreatic	Alanine aminotransaminase	Diagnosis		Joinpoint regression for cases and controls separately	Method: Joinpoint regression for cases and controls separately. Result: Case+recent T2D: time -60 to -9 coef=-0.08, time -9 to -3 coef=14.18; Case+long-standing T2D: -60 to -9 coef=-0.12, time -9 to -3 coef=14.25; Case+no T2D: time -60 to -9 coef=-0.00, time -9 to -3 coef=18.14; Control+recent T2D: time -60 to -39 coef=0.08, time -39 to -3 coef=-0.09; Control+long-standing T2D: time -60 to -3 coef=-0.07; Control+no T2D: time -60 to -3 coef=-0.03	Tan 2023
Pancreatic	Aspartate transaminase	Diagnosis		Joinpoint regression for cases and controls separately	Method: Joinpoint regression for cases and controls separately. Result: Case+recent T2D: time -60 to -9 coef=0.08, time -9 to -3 coef=10.65; Case+long-standing T2D: -60 to -9 coef=-0.03, time -9 to -3 coef=12.13; Case+no T2D: time -60 to -9 coef=0.03, time -9 to -3 coef=13.00; Control+recent T2D: time -60 to -51 coef=-0.24, time -51 to -42 coef=0.49, time -42 to -3 coef=-0.07; Control+long-standing T2D: time -60 to -3 coef=-0.01; Control+no T2D: time -60 to -3 coef=-0.00	Tan 2023
Pancreatic	Bilirubin	Diagnosis		Joinpoint regression for cases and controls separately	Method: Joinpoint regression for cases and controls separately. Result: Case+recent T2D: time -60 to -9 coef=-0.03, time -9 to -3 coef=5.45; Case+long-standing T2D: -60 to -9 coef=-0.04, time -9 to -3	Tan 2023

					coef=5.41; Case+no T2D: time -60 to -9 coef=-0.04, time -9 to -3 coef=6.91; Control+recent T2D: time -60 to -9 coef=-0.00, time -9 to -3 coef=0.16; Control+long-standing T2D: time -60 to -9 coef=-0.01, time -9 to -3 coef=0.22; Control+no T2D: time -60 to -9 coef=-0.01, time -9 to -3 coef=0.40	
Pancreatic	Fasting glucose	-0-6m		T-test stratified by time	Group 1=Cancer (n=159, mean=134), Group 2=No cancer (n=123, mean=108), Results=p<0.001	Sharma 2018
Pancreatic	Fasting glucose	-12-18m		T-test stratified by time	Group 1=Cancer (n=90, mean=118), Group 2=No cancer (n=123, mean=106), Results=p<0.001	Sharma 2018
Pancreatic	Fasting glucose	-18-24m		T-test stratified by time	Group 1=Cancer (n=69, mean=115), Group 2=No cancer (n=86, mean=106), Results=p=0.02	Sharma 2018
Pancreatic	Fasting glucose	-24-30m		T-test stratified by time	Group 1=Cancer (n=71, mean=114), Group 2=No cancer (n=128, mean=105), Results=p=0.009	Sharma 2018
Pancreatic	Fasting glucose	-30-36m		T-test stratified by time	Group 1=Cancer (n=68, mean=112), Group 2=No cancer (n=116, mean=104), Results=p=0.01	Sharma 2018
Pancreatic	Fasting glucose	-36-42m		T-test stratified by time	Group 1=Cancer (n=61, mean=106), Group 2=No cancer (n=125, mean=105), Results=p=0.62	Sharma 2018
Pancreatic	Fasting glucose	-42-48m		T-test stratified by time	Group 1=Cancer (n=60, mean=105), Group 2=No cancer (n=99, mean=104), Results=p=0.57	Sharma 2018
Pancreatic	Fasting glucose	-48-54m		T-test stratified by time	Group 1=Cancer (n=72, mean=104), Group 2=No cancer (n=122, mean=105), Results=p=0.70	Sharma 2018
Pancreatic	Fasting glucose	-54-60m		T-test stratified by time	Group 1=Cancer (n=81, mean=103), Group 2=No cancer (n=106, mean=103), Results=p=0.91	Sharma 2018
Pancreatic	Fasting glucose	-6-12m		T-test stratified by time	Group 1=Cancer (n=88, mean=127), Group 2=No cancer (n=93, mean=106), Results=p<0.001	Sharma 2018

Pancreatic	Fasting glucose	3 years		Generalised estimating equation	Method: Generalised estimating equation. Result: Having adjusted for age, sex, and race as covariates and patient as the clustering variable, the slope from 3yr to 1m before diabetes diagnosis in cases=1.19 (0.71 to 1.67) and non-cases=0.81 (0.78 to 0.84). P=values for differences in slopes between cases and controls taken from a third GEE model including a time*case status interaction, p=0.06. Results did not differ much when assessed in subgroups of white, asian, black, hispanic.	Huang 2020
Pancreatic	Fasting glucose	Diagnosis		Linear regression for cases and controls separately	Method: Linear regression for cases and controls separately. Result: Cases: FGB=99.71+(5.08*time); controls=FGB=100.39+(0.40*time)	Pannala 2009
Pancreatic	Fasting glucose	Diagnosis		Linear regression per time interval	Method: Linear regression per time interval. Result: No model results were reported - only the predicted trend graphically	Sharma 2018
Pancreatic	Haemoglobin	Diagnosis		Joinpoint regression for cases and controls separately	Method: Joinpoint regression for cases and controls separately. Result: Case+recent T2D: time -60 to -24 coef=-0.01, time -24 to -9 coef=-0.02, time -9 to -3 coef=-0.11; Case+long-standing T2D: -60 to -9 coef=-0.01, time -9 to -3 coef=-0.11; Case+no T2D: time -60 to -21 coef=-0.00, time -21 to -9 coef=-0.02, time -9 to -3 coef=-0.10; Control+recent T2D: time -60 to -33 coef=-0.00, time -33 to -3 coef=-0.01; Control+long-standing T2D: time -60 to -54 coef=-0.00, time -54 to -9 coef=-0.01, time -9 to -3 coef=-0.02; Control+no T2D: time -60 to -27 coef=-0.00, time -27 to -3 coef=-0.01	Tan 2023
Pancreatic	HbA1C	-0-6 months		Logistic regression per time-point	Group 1 (Ref)=27 – 45; n=144, cases=6. Group 2=46 – 51; n=166, cases=14, OR=2.18 (0.80 – 5.98). Group 3=52 – 60; n=150, cases=10, OR=1.46 (0.51 – 4.23).	Sadr-Azodi 2015

					Group 4=61 – 145; n=167, cases=27, OR=4.73 (1.83 – 12.21). Multivariable, adjusted for: Education, smoking, body mass index, alcohol abuse, complicated diabetes disease, previous pancreatitis	
Pancreatic	HbA1C	-1-2 years		Logistic regression per time-point	Group 1 (Ref)=27 – 45; n=186, cases=11. Group 2=46 – 51; n=198, cases=16, OR=1.37 (0.61 – 3.11). Group 3=52 – 60; n=192, cases=17, OR=1.56 (0.69 – 3.52). Group 4=61 – 145; n=183, cases=25, OR=2.51 (1.17 – 5.40). Multivariable, adjusted for: Education, smoking, body mass index, alcohol abuse, complicated diabetes disease, previous pancreatitis	Sadr-Azodi 2015
Pancreatic	HbA1C	-2-5 years		Logistic regression per time-point	Group 1 (Ref)=27 – 45; n=338, cases=21. Group 2=46 – 51; n=395, cases=36, OR=1.52 (0.86 – 2.70). Group 3=52 – 60; n=444, cases=47, OR=1.86 (1.07 – 3.25). Group 4=61 – 145; n=394, cases=39, OR=1.77 (0.99 – 3.19). Multivariable, adjusted for: Education, smoking, body mass index, alcohol abuse, complicated diabetes disease, previous pancreatitis	Sadr-Azodi 2015
Pancreatic	HbA1C	-5+ years		Logistic regression per time-point	Group 1 (Ref)=27 – 45; n=141, cases=14. Group 2=46 – 51; n=151, cases=15, OR=0.91 (0.41 – 2.02). Group 3=52 – 60; n=232, cases=23, OR=0.98 (0.47 – 2.04). Group 4=61 – 145; n=333, cases=26, OR=0.69 (0.32 – 1.48). Multivariable, adjusted for: Education, smoking, body mass index, alcohol abuse, complicated diabetes disease, previous pancreatitis	Sadr-Azodi 2015
Pancreatic	HbA1C	-6-12 months		Logistic regression per time-point	Group 1 (Ref)=27 – 45; n=104, cases=6. Group 2=46 – 51; n=131, cases=7, OR=0.87 (0.27 – 2.80). Group 3=52 – 60; n=132, cases=15, OR=2.24 (0.82 – 6.13). Group 4=61 – 145; n=117, cases=16, OR=2.70 (0.99 – 7.36). Multivariable, adjusted for: Education,	Sadr-Azodi 2015

					smoking, body mass index, alcohol abuse, complicated diabetes disease, previous pancreatitis	
Pancreatic	HbA1C	3 years		Generalised estimating equation	Method: Generalised estimating equation. Result: Having adjusted for age, sex, and race as covariates and patient as the clustering variable, the slope from 3yr to 1m before diabetes diagnosis in cases=0.03 (0.00 to 0.06) and non-cases=0.04 (0.04 to 0.04). P=values for differences in slopes between cases and controls taken from a third GEE model including a time*case status interaction, p=0.38. Results did not differ much when assessed in subgroups of white, asian, black, hispanic.	Huang 2020
Pancreatic	HbA1C	Diagnosis		Joinpoint regression for cases and controls separately	Method: Joinpoint regression for cases and controls separately. Result: Case+recent T2D: time -60 to -42 coef=0.16, time -42 to -33 coef=0.78, time -33 to -21 coef=-0.12, time -21 to -3 coef=0.46; Case+long-standing T2D: -60 to -33 coef=0.04, time -33 to -18 coef=0.16, time -18 to -3=0.42; Case+no T2D: time -60 to -33 coef=-0.04, time -33 to -18 coef=0.09, time -18 to -9=0.21, time -9 to -3 coef=0.40; Control+recent T2D: time -60 to -45 coef=0.08, time -45 to -36 coef=0.71, time -36 to -3 coef=-0.07; Control+long-standing T2D: time -60 to -3 coef=0.01; Control+no T2D: time -60 to -30 coef=-0.02, time -30 to -3 coef=0.01	Tan 2023
Pancreatic	HbA1C	Diagnosis		Logistic regression per time-point	n=43756, cases=8777, OR=Diagnosis=1.18 (1.17 to 1.19), p<0.001; -1y=1.05 (1.05 to 1.06), p<0.001; -2y=1.01 (1.01 to 1.02), p<0.001; -3y=1.00 (1.00 to 1.01), p=0.226; -4y=1.00 (1.00 to 1.01), p=0.936; -4y=1.00 (0.99 to 1.00), p=0.614. Multivariable, adjusted for: Age, sex, diabetes	Lemanska 2022

Pancreatic	Platelet count	Diagnosis		Joinpoint regression for cases and controls separately	Method: Joinpoint regression for cases and controls separately. Result: Case+recent T2D: time -60 to -9 coef=-0.16, time -9 to -3 coef=2.82; Case+long-standing T2D: -60 to -9 coef=-0.02, time -9 to -3 coef=4.23; Case+no T2D: time -60 to -9 coef=-0.06, time -9 to -3 coef=4.06; Control+recent T2D: time -60 to -3 coef=-0.13; Control+long-standing T2D: time -60 to -15 coef=-0.02, time -15 to -3 coef=0.13; Control+no T2D: time -60 to -9 coef=-0.05, time -9 to -3 coef=0.41	Tan 2023
Pancreatic	White blood cell count	Diagnosis		Joinpoint regression for cases and controls separately	Method: Joinpoint regression for cases and controls separately. Result: Case+recent T2D: time -60 to -9 coef=0.00, time -9 to -3 coef=0.13; Case+long-standing T2D: -60 to -9 coef=-0.00, time -9 to -3 coef=-0.16; Case+no T2D: time -60 to -9 coef=0.00, time -9 to -3 coef=0.19; Control+recent T2D: time -60 to -24 coef=0.01, time -24 to -3 coef=-0.00; Control+long-standing T2D: time -60 to -3 coef=0.00; Control+no T2D: time -60 to -9 coef=0.00, time -9 to -3 coef=0.02	Tan 2023
Prostate	Haemoglobin	-1 year		Logistic regression stratified by time	OR=0.97 (0.89–1.05)	Edgren 2010
Prostate	Haemoglobin	-1-2 years		AUC stratified by time	AUC=0.53 (0.50-0.55)	Edgren 2010
Prostate	Haemoglobin	-1-2 years		Logistic regression stratified by time	OR=1.00 (0.93–1.08)	Edgren 2010
Prostate	Haemoglobin	-2-3 years		Logistic regression stratified by time	OR=1.00 (0.93–1.08)	Edgren 2010

Prostate	Haemoglobin	-3-4 years		Logistic regression stratified by time	OR=0.99 (0.92–1.07)	Edgren 2010
Prostate	Haemoglobin	-4-5 years		Logistic regression stratified by time	OR=0.98 (0.91–1.05)	Edgren 2010
Renal cell	Erythrocyte sedimentation rate	Diagnosis		Linear regression including repeated blood tests	Method: Linear regression including repeated blood tests. Result: No coefficients were presented. They only presented the linear regression line of best fit for cases and controls separately	Iversen 1996
Small intestine	Haemoglobin	-1 year		Logistic regression stratified by time	OR=1.59 (1.05–2.39)	Edgren 2010
Small intestine	Haemoglobin	-1-2 years		AUC stratified by time	AUC=0.56 (0.45-0.67)	Edgren 2010
Small intestine	Haemoglobin	-1-2 years		Logistic regression stratified by time	OR=1.56 (1.04–2.34)	Edgren 2010
Small intestine	Haemoglobin	-2-3 years		Logistic regression stratified by time	OR=0.97 (0.66–1.43)	Edgren 2010
Small intestine	Haemoglobin	-3-4 years		Logistic regression stratified by time	OR=0.87 (0.59–1.28)	Edgren 2010
Small intestine	Haemoglobin	-4-5 years		Logistic regression stratified by time	OR=1.17 (0.78–1.74)	Edgren 2010
Stomach	Haemoglobin	-1 year		Logistic regression stratified by time	OR=1.57 (1.31–1.88)	Edgren 2010
Stomach	Haemoglobin	-1-2 years		AUC stratified by time	AUC=0.59 (0.55-0.64)	Edgren 2010
Stomach	Haemoglobin	-1-2 years		Logistic regression stratified by time	OR=1.32 (1.11–1.58)	Edgren 2010

Stomach	Haemoglobin	-2-3 years		Logistic regression stratified by time	OR=1.29 (1.09–1.54)	Edgren 2010
Stomach	Haemoglobin	-3-4 years		Logistic regression stratified by time	OR=1.09 (0.92–1.29)	Edgren 2010
Stomach	Haemoglobin	-4-5 years		Logistic regression stratified by time	OR=1.08 (0.91–1.28)	Edgren 2010
Thyroid	Thyroid Stimulating Hormone	Diagnosis		Unclear	Method: Unclear. Result: Mean TSH over time does not statistically significantly differ in cases up to diagnosis (p=0.57). This was not assessed in controls	Rinaldi 2014

[1]Risk window is the outcome time frame. "Diagnosis" means studied analysed trends until diagnosis. Positive numbers mean the study analysed trend and predicted subsequent cancer risk, e.g. "3 years" means three-year risk following trend. Negative numbers mean the study analysed trend prior to diagnosis, e.g. "-1-2 years" means 1-2 years of trend prior to diagnosis.

Table S8: Cancer outcome details per study

Article	Blood test trend analysed	Cancer outcome	Risk window start	Risk window end	Number of patients diagnosed
Atkin 2020	Calcium adjusted, Creatinine, Haematocrit, Haemoglobin	Multiple myeloma=yes	Diagnosis	Diagnosis	yes=285
Boursi 2016	Albumin, Blood glucose, Calcium, Haematocrit, Mean cell haemoglobin, Mean cell volume, Red blood cell count, White blood cell count	Colorectal=yes/no	Diagnosis	Unclear	yes=9299; no=36199
Chaturvedi 2010	C-reactive protein	Lung=yes/no	First blood test	1 year	
			First blood test	1-2 years	
			First blood test	2-5 years	
			First blood test	5+ years	
			First blood test	Diagnosis	yes=670; no=592
Edgren 2010	Haemoglobin	Cancer=yes/no	Diagnosis	Diagnosis	yes=16375; no=161995
			Diagnosis	-1 year	
			Diagnosis	-1-2 years	
			Diagnosis	-2-3 years	
			Diagnosis	-3-4 years	
			Diagnosis	-4-5 years	
		Breast=yes/no	Diagnosis	-1 year	

			Diagnosis	-1-2 years	
			Diagnosis	-2-3 years	
			Diagnosis	-3-4 years	
			Diagnosis	-4-5 years	
		Colon=yes/no	Diagnosis	-1 year	
			Diagnosis	-1-2 years	
			Diagnosis	-2-3 years	
			Diagnosis	-3-4 years	
			Diagnosis	-4-5 years	
		Hodgkin lymphoma=yes/no	Diagnosis	-1 year	
			Diagnosis	-1-2 years	
			Diagnosis	-2-3 years	
			Diagnosis	-3-4 years	
			Diagnosis	-4-5 years	
		Lymphatic leukemia=yes/no	Diagnosis	-1 year	
			Diagnosis	-1-2 years	
			Diagnosis	-2-3 years	
			Diagnosis	-3-4 years	
			Diagnosis	-4-5 years	
		Multiple myeloma=yes/no	Diagnosis	-1 year	

			Diagnosis	-1-2 years	
			Diagnosis	-2-3 years	
			Diagnosis	-3-4 years	
			Diagnosis	-4-5 years	
		Myeloid/monocytic leukemia=yes/no	Diagnosis	-1 year	
			Diagnosis	-1-2 years	
			Diagnosis	-2-3 years	
			Diagnosis	-3-4 years	
			Diagnosis	-4-5 years	
		Non-Hodgkin lymphoma=yes/no	Diagnosis	-1 year	
			Diagnosis	-1-2 years	
			Diagnosis	-2-3 years	
			Diagnosis	-3-4 years	
			Diagnosis	-4-5 years	
		Oesophagus=yes/no	Diagnosis	-1 year	
			Diagnosis	-1-2 years	
			Diagnosis	-2-3 years	
			Diagnosis	-3-4 years	
			Diagnosis	-4-5 years	
		Prostate=yes/no	Diagnosis	-1 year	

			Diagnosis	-1-2 years	
			Diagnosis	-2-3 years	
			Diagnosis	-3-4 years	
			Diagnosis	-4-5 years	
		Small intestine=yes/no	Diagnosis	-1 year	
			Diagnosis	-1-2 years	
			Diagnosis	-2-3 years	
			Diagnosis	-3-4 years	
			Diagnosis	-4-5 years	
		Stomach=yes/no	Diagnosis	-1 year	
			Diagnosis	-1-2 years	
			Diagnosis	-2-3 years	
			Diagnosis	-3-4 years	
			Diagnosis	-4-5 years	
Feng 2020	Fasting glucose	Cancer=yes/no	Last blood test	6 years	yes=1364; no=68378
		GI cancer=yes/no	Last blood test	6 years	yes=472; no=69270
Fuente 2019	Monocyte count	Pancreatic=yes/no	Diagnosis	Diagnosis	yes=219; no=438
Furukawa 1984	Aspartate transaminase	Liver=yes/no	Unclear	Diagnosis	yes=29; no=85
Giannakeas 2022	Platelet count	Breast=yes	Diagnosis	Diagnosis	yes=57012
		Colon=yes	Diagnosis	Diagnosis	yes=45461

		Lung=yes	Diagnosis	Diagnosis	yes=71427
		Ovarian=yes	Diagnosis	Diagnosis	yes=6451
		Prostate=yes	Diagnosis	Diagnosis	yes=55567
		Stomach=yes	Diagnosis	Diagnosis	yes=7118
Goldshtein 2010	Haemoglobin	Colorectal=yes/no	Diagnosis	Diagnosis	yes=1074; no=10740
Gradel 2020	Albumin, C-reactive protein	Acute myeloid leukaemia=yes	Diagnosis	Diagnosis	
Hauser 2021	Basophil %	Chronic myelogenous leukemia=yes/no	Diagnosis	Diagnosis	yes=100; no=1523
Hsieh 2019	Platelet count	Hematologic malignancy=yes	Unclear	Unclear	yes=85
Huang 2020	Fasting glucose, HbA1C	Pancreatic=yes/no	Study entry	3 years	yes=306; no=110393
Iversen 1996	Erythrocyte sedimentation rate	Renal cell=yes/no	Diagnosis	Diagnosis	yes=236; no=3910
Jacobson 2021	Fasting glucose	Pancreatic=yes/no	Diagnosis	Diagnosis	yes=182; no=717
Jonsson 2020	Creatinine, Urea	Glioma=yes/no	Diagnosis	Diagnosis	yes=64; no=64
Koshiaris 2018	C-reactive protein, Calcium, Creatinine, Erythrocyte sedimentation rate, Haemoglobin, Mean cell volume, Plasma viscosity	Multiple myeloma=yes/no	Diagnosis	Diagnosis	yes=2703; no=12157
Kubo 2016	Alanine aminotransaminase, Aspartate transaminase	Cholangiocarcinoma (bile duct)=yes	Diagnosis	Diagnosis	yes=9
Lemanska 2022	HbA1C	Pancreatic=yes/no	Diagnosis	Diagnosis	yes=8777; no=34979
Li 2021	Haemoglobin	Colorectal=yes/no	Diagnosis	Diagnosis	yes=1534; no=15333
Pannala 2009	Fasting glucose	Pancreatic=yes/no	Diagnosis	Diagnosis	yes=736; no=1875

Rinaldi 2014	Thyroid Stimulating Hormone	Thyroid=yes/no	Diagnosis	Diagnosis	yes=357; no=767
Sadr-Azodi 2015	HbA1C	Pancreatic=yes/no	Diagnosis	-0-6 months	yes=57; no=570
			Diagnosis	-1-2 years	yes=69; no=690
			Diagnosis	-2-5 years	yes=143; no=1428
			Diagnosis	-5+ years	yes=78; no=779
			Diagnosis	-6-12 months	yes=44; no=440
Sharma 2018	Fasting glucose	Pancreatic=yes/no	Diagnosis	Diagnosis	yes=219; no=440
			Diagnosis	-0-6m	yes=159; no=123
			Diagnosis	-6-12m	yes=88; no=93
			Diagnosis	-12-18m	yes=90; no=123
			Diagnosis	-18-24m	yes=69; no=86
			Diagnosis	-24-30m	yes=71; no=128
			Diagnosis	-30-36m	yes=68; no=116
			Diagnosis	-36-42m	yes=61; no=125
			Diagnosis	-42-48m	yes=60; no=99
			Diagnosis	-48-54m	yes=72; no=122
			Diagnosis	-54-60m	yes=81; no=106
Stroud 2020	Blood glucose	Cancer=yes/no	First blood test	7 years	yes=82; no=2025
Tan 2023	Alanine aminotransaminase, Aspartate transaminase, Bilirubin, Haemoglobin, HbA1C,	Pancreatic=yes/no	Diagnosis	Diagnosis	yes=28137; no=261219

	Platelet count, White blood cell count				
Toriola 2011	C-reactive protein	Ovarian=yes	Diagnosis	Diagnosis	yes=170; no=170
Toriola 2013	C-reactive protein	Colorectal=yes/no	First blood test	Unclear	yes=766; no=875
Virdee 2022	Basophil count, Eosinophil count, Haematocrit, Haemoglobin, Lymphocyte count, Mean cell haemoglobin, Mean cell haemoglobin concentration, Mean cell volume, Mean platelet volume, Monocyte count, Neutrophil count, Platelet count, Red blood cell count, White blood cell count	Colorectal=yes/no	Diagnosis	Diagnosis	yes=17408; no=541646

Table S9: Description of graphical blood test trend by cancer site

Cancer site	Risk window[1]	Blood level	Strata (if applicable)	Description of graphical trend	Article
Acute myeloid leukaemia	Diagnosis	Albumin		Cases: remained steady	Gradel 2020
Acute myeloid leukaemia	Diagnosis	C-reactive protein		Cases: remained steady (only trends within 30 days before diagnosis shown)	Gradel 2020
Breast	Diagnosis	Haemoglobin		No difference between cases and non-cases	Edgren 2010
Breast	Diagnosis	Platelet count		Cases: remained steady	Giannakeas 2022
Cancer	Diagnosis	Haemoglobin		Cases: decline at 1 year before diagnosis. Non-cases: remained steady	Edgren 2010
Cholangiocarcinoma (bile duct)	Diagnosis	Alanine aminotransaminase		Cases: increases slightly at 2 years before diagnosis	Kubo 2016
Cholangiocarcinoma (bile duct)	Diagnosis	Aspartate transaminase		Cases: increases slightly at 2 years before diagnosis	Kubo 2016
Chronic myelogenous leukemia	Diagnosis	Basophil %		Cases: increases slightly at 1 year before diagnosis	Hauser 2021
Colon	Diagnosis	Haemoglobin		Cases: decline at 2 year before diagnosis. Non-cases: remained steady	Edgren 2010
Colon	Diagnosis	Platelet count		Cases: increases at 1 year before diagnosis, with an increasing rate of change as diagnosis approaches	Giannakeas 2022
Colorectal	Diagnosis	Basophil count	Females	No difference between cases and non-cases	Virdee 2022
Colorectal	Diagnosis	Basophil count	Males	No difference between cases and non-cases	Virdee 2022
Colorectal	Diagnosis	Eosinophil count	Females	No difference between cases and non-cases	Virdee 2022

Colorectal	Diagnosis	Eosinophil count	Males	No difference between cases and non-cases	Virdee 2022
Colorectal	Diagnosis	Haematocrit/Packed cell volume	Females	Cases: declined at 3 years before diagnosis, with an increasing rate of decline as diagnosis approaches. Non-cases: remained steady	Virdee 2022
Colorectal	Diagnosis	Haematocrit/Packed cell volume	Males	Cases: declined at 3 years before diagnosis, with an increasing rate of decline as diagnosis approaches. Non-cases: remained steady	Virdee 2022
Colorectal	Diagnosis	Haemoglobin		Cases: declines at 4 years before diagnosis, with an increasing rate of decline as diagnosis approaches. Non-cases: remained steady	Goldshtein 2010
Colorectal	Diagnosis	Haemoglobin		Cases: decline at 2-3 years before diagnosis, with an increasing rate of decline as diagnosis approached. Non-cases: remained steady	Li 2021
Colorectal	Diagnosis	Haemoglobin	Females	Cases: declined at 5 years before diagnosis, with an increasing rate of decline as diagnosis approaches. Non-cases: remained steady	Virdee 2022
Colorectal	Diagnosis	Haemoglobin	Males	Cases: declined at 5 years before diagnosis, with an increasing rate of decline as diagnosis approaches. Non-cases: remained steady	Virdee 2022
Colorectal	Diagnosis	Lymphocyte count	Females	Cases: declines slightly at 6 months before diagnosis. Non-cases: remained steady	Virdee 2022
Colorectal	Diagnosis	Lymphocyte count	Males	Cases: declines slightly at 6 months before diagnosis. Non-cases: remained steady	Virdee 2022
Colorectal	Diagnosis	Mean cell haemoglobin	Females	Cases: declined at 3 years before diagnosis, with an increasing rate of decline as diagnosis approaches. Non-cases: remained steady	Virdee 2022
Colorectal	Diagnosis	Mean cell haemoglobin	Males	Cases: declined at 3 years before diagnosis, with an increasing rate of decline as diagnosis approaches. Non-cases: remained steady	Virdee 2022
Colorectal	Diagnosis	Mean cell haemoglobin concentration	Females	Cases: declined at 3 years before diagnosis, with an increasing rate of decline as diagnosis approaches. Non-cases: remained steady	Virdee 2022

Colorectal	Diagnosis	Mean cell haemoglobin concentration	Males	Cases: declined at 3 years before diagnosis, with an increasing rate of decline as diagnosis approaches. Non-cases: remained steady	Virdee 2022
Colorectal	Diagnosis	Mean cell volume	Females	Cases: declined at 4 years before diagnosis, with an increasing rate of decline as diagnosis approaches. Non-cases: remained steady	Virdee 2022
Colorectal	Diagnosis	Mean cell volume	Males	Cases: declined at 4 years before diagnosis, with an increasing rate of decline as diagnosis approaches. Non-cases: remained steady	Virdee 2022
Colorectal	Diagnosis	Mean platelet volume	Females	Cases: declines slightly at 6 months before diagnosis. Non-cases: remained steady	Virdee 2022
Colorectal	Diagnosis	Mean platelet volume	Males	Cases: declines slightly at 6 months before diagnosis. Non-cases: remained steady	Virdee 2022
Colorectal	Diagnosis	Monocyte count	Females	Cases: increases at 1 year before diagnosis, with an increasing rate of change as diagnosis approaches. Non-cases: remained steady	Virdee 2022
Colorectal	Diagnosis	Monocyte count	Males	Cases: increases at 1 year before diagnosis, with an increasing rate of change as diagnosis approaches. Non-cases: remained steady	Virdee 2022
Colorectal	Diagnosis	Neutrophil count	Females	Cases: increases at 3 years before diagnosis, with an increasing rate of change as diagnosis approaches. Non-cases: remained steady	Virdee 2022
Colorectal	Diagnosis	Neutrophil count	Males	Cases: increases at 3 years before diagnosis, with an increasing rate of change as diagnosis approaches. Non-cases: remained steady	Virdee 2022
Colorectal	Diagnosis	Platelet count	Females	Cases: increases at 4 years before diagnosis, with an increasing rate of change as diagnosis approaches. Non-cases: remained steady	Virdee 2022
Colorectal	Diagnosis	Platelet count	Males	Cases: increases at 4 years before diagnosis, with an increasing rate of change as diagnosis approaches. Non-cases: remained steady	Virdee 2022
Colorectal	Diagnosis	Red blood cell count	Females	Cases: declined at 3 years before diagnosis, with an increasing rate of decline as diagnosis approaches. Non-cases: remained steady	Virdee 2022

Colorectal	Diagnosis	Red blood cell count	Males	Cases: declined at 3 years before diagnosis, with an increasing rate of decline as diagnosis approaches. Non-cases: remained steady	Virdee 2022
Colorectal	Diagnosis	White blood cell count	Females	Cases: increases at 2 years before diagnosis, with an increasing rate of change as diagnosis approaches. Non-cases: remained steady	Virdee 2022
Colorectal	Diagnosis	White blood cell count	Males	Cases: increases at 2 years before diagnosis, with an increasing rate of change as diagnosis approaches. Non-cases: remained steady	Virdee 2022
Hematologic malignancy	Unclear	Platelet count		Cases: remained steady	Hsieh 2019
Hodgkin lymphoma	Diagnosis	Haemoglobin		Cases: decline at 4 years before diagnosis. Non-cases: remained steady	Edgren 2010
Lung	Diagnosis	Platelet count		Cases: increases at 9 months before diagnosis, with an increasing rate of change at 3 months before diagnosis	Giannakeas 2022
Lymphatic leukemia	Diagnosis	Haemoglobin		Cases: decline at 3 years before diagnosis. Non-cases: remained steady	Edgren 2010
Multiple myeloma	Diagnosis	C-reactive protein		No difference between cases and non-cases	Koshiaris 2018
Multiple myeloma	Diagnosis	Calcium		Cases: increases slightly at 1 year before diagnosis, with an increasing rate of change at 6 months before diagnosis. Non-cases: remained steady	Koshiaris 2018
Multiple myeloma	Diagnosis	Calcium adjusted		Cases: increases at 90 days before diagnosis	Atkin 2020
Multiple myeloma	Diagnosis	Creatinine		Cases: increases at 90 days before diagnosis	Atkin 2020
Multiple myeloma	Diagnosis	Creatinine		Cases: increases slightly at 1 year before diagnosis, with an increasing rate of change at 6 months before diagnosis. Non-cases: remained steady	Koshiaris 2018
Multiple myeloma	Diagnosis	Erythrocyte sedimentation rate		Cases: increases slightly at 2 years before diagnosis, with an increasing rate of change at 9 months before diagnosis. Non-cases: remained steady	Koshiaris 2018
Multiple myeloma	Diagnosis	Haematocrit/Packed cell volume		Cases: decline at 2 years before diagnosis	Atkin 2020

Multiple myeloma	Diagnosis	Haemoglobin		Cases: decline at 2 years before diagnosis	Atkin 2020
Multiple myeloma	Diagnosis	Haemoglobin		Cases: decline at 4 years before diagnosis, with an increasing rate of decline from 1 year before diagnosis. Non-cases: remained steady	Edgren 2010
Multiple myeloma	Diagnosis	Haemoglobin	Females	Cases: decline at 3 years before diagnosis, with an increasing rate of decline as diagnosis appproached. Non-cases: remained steady	Koshiaris 2018
Multiple myeloma	Diagnosis	Haemoglobin	Males	Cases: decline at 3 years before diagnosis, with an increasing rate of decline as diagnosis appproached. Non-cases: remained steady	Koshiaris 2018
Multiple myeloma	Diagnosis	Mean cell volume		Cases: increases slightly at 1 year before diagnosis. Non-cases: remained steady	Koshiaris 2018
Multiple myeloma	Diagnosis	Plasma viscosity		Cases: increases slightly at 1 year before diagnosis, with an increasing rate of change as diagnosis approached. Non-cases: remained steady	Koshiaris 2018
Myeloid/monocytic leukemia	Diagnosis	Haemoglobin		Cases: decline at 1 year before diagnosis. Non-cases: remained steady	Edgren 2010
Non-Hodgkin lymphoma	Diagnosis	Haemoglobin		Cases: decline at 3 years before diagnosis. Non-cases: remained steady	Edgren 2010
Oesophagus	Diagnosis	Haemoglobin		Cases: decline at 5 year before diagnosis. Non-cases: remained steady	Edgren 2010
Ovarian	Diagnosis	Platelet count		Cases: increases at 9 months before diagnosis, with an increasing rate of change at 3 months before diagnosis	Giannakeas 2022
Pancreatic	-0-6 months	HbA1C		Cases: increases at 2-3 years before diagnosis. Non-cases: remained steady	Sadr-Azodi 2015
Pancreatic	-0-6m	Fasting glucose		Cases: increases at 3 years before diagnosis, with an increasing rate of change at 1 year before diagnosis. Non-cases: remained steady	Sharma 2018
Pancreatic	-1-2 years	HbA1C		Cases: increases at 2-3 years before diagnosis. Non-cases: remained steady	Sadr-Azodi 2015

Pancreatic	-12-18m	Fasting glucose		Cases: increases at 3 years before diagnosis, with an increasing rate of change at 1 year before diagnosis. Non-cases: remained steady	Sharma 2018
Pancreatic	-18-24m	Fasting glucose		Cases: increases at 3 years before diagnosis, with an increasing rate of change at 1 year before diagnosis. Non-cases: remained steady	Sharma 2018
Pancreatic	-2-5 years	HbA1C		Cases: increases at 2-3 years before diagnosis. Non-cases: remained steady	Sadr-Azodi 2015
Pancreatic	-24-30m	Fasting glucose		Cases: increases at 3 years before diagnosis, with an increasing rate of change at 1 year before diagnosis. Non-cases: remained steady	Sharma 2018
Pancreatic	-30-36m	Fasting glucose		Cases: increases at 3 years before diagnosis, with an increasing rate of change at 1 year before diagnosis. Non-cases: remained steady	Sharma 2018
Pancreatic	-36-42m	Fasting glucose		Cases: increases at 3 years before diagnosis, with an increasing rate of change at 1 year before diagnosis. Non-cases: remained steady	Sharma 2018
Pancreatic	-42-48m	Fasting glucose		Cases: increases at 3 years before diagnosis, with an increasing rate of change at 1 year before diagnosis. Non-cases: remained steady	Sharma 2018
Pancreatic	-48-54m	Fasting glucose		Cases: increases at 3 years before diagnosis, with an increasing rate of change at 1 year before diagnosis. Non-cases: remained steady	Sharma 2018
Pancreatic	-5+ years	HbA1C		Cases: increases at 2-3 years before diagnosis. Non-cases: remained steady	Sadr-Azodi 2015
Pancreatic	-54-60m	Fasting glucose		Cases: increases at 3 years before diagnosis, with an increasing rate of change at 1 year before diagnosis. Non-cases: remained steady	Sharma 2018
Pancreatic	-6-12 months	HbA1C		Cases: increases at 2-3 years before diagnosis. Non-cases: remained steady	Sadr-Azodi 2015
Pancreatic	-6-12m	Fasting glucose		Cases: increases at 3 years before diagnosis, with an increasing rate of change at 1 year before diagnosis. Non-cases: remained steady	Sharma 2018

Pancreatic	3 years	Fasting glucose		Cases and non-cases: increases slightly, with a rapid increase at 6 months before diabetes diagnosis, although cases had a higher increase at 6 months before diabetes diagnosis	Huang 2020
Pancreatic	3 years	HbA1C		Cases and non-cases: increases slightly, with a rapid increase at 6 months before diabetes diagnosis, although cases had a higher increase at 6 months before diabetes diagnosis	Huang 2020
Pancreatic	Diagnosis	Alanine aminotransaminase		Cases: increases at 6-12 months before diagnosis. Non-cases: remained steady	Tan 2023
Pancreatic	Diagnosis	Aspartate transaminase		Cases: increases at 6-12 months before diagnosis. Non-cases: remained steady	Tan 2023
Pancreatic	Diagnosis	Bilirubin		Cases: increases at 6-12 months before diagnosis. Non-cases: remained steady	Tan 2023
Pancreatic	Diagnosis	Fasting glucose		Cases: increases at 6 years before diagnosis, with an increasing rate of change 1 year before diagnosis	Jacobson 2021
Pancreatic	Diagnosis	Fasting glucose		Cases: increases at 4 years before diagnosis, with an increasing rate of change at 1.5 years before diagnosis. Non-cases: remained steady	Pannala 2009
Pancreatic	Diagnosis	Fasting glucose		Cases: increases at 3 years before diagnosis, with an increasing rate of change at 1 year before diagnosis. Non-cases: remained steady	Sharma 2018
Pancreatic	Diagnosis	Haemoglobin		Cases: declines at 2 years before diagnosis, with an increasing rate of change as diagnosis approaches. Non-cases: remained steady	Tan 2023
Pancreatic	Diagnosis	HbA1C		Cases: increases at 2 years before diagnosis. Non-cases: remained steady	Lemanska 2022
Pancreatic	Diagnosis	HbA1C		Cases: increases at 3 years before diagnosis, with an increasing rate of change as diagnosis approaches. Non-cases: remained steady	Tan 2023
Pancreatic	Diagnosis	Monocyte count		Cases: increase at 1 year before diagnosis. Non-cases: remained steady	Fuente 2019
Pancreatic	Diagnosis	Platelet count		Cases: increases at 6-12 months before diagnosis. Non-cases: remained steady	Tan 2023

Pancreatic	Diagnosis	White blood cell count		Cases: increases at 6-12 months before diagnosis. Non-cases: remained steady	Tan 2023
Prostate	Diagnosis	Haemoglobin		No difference between cases and non-cases	Edgren 2010
Prostate	Diagnosis	Platelet count		Cases: remained steady	Giannakeas 2022
Renal cell	Diagnosis	Erythrocyte sedimentation rate		Cases: increases at 5 years before diagnosis, with a rapid increase at 1 year before diagnosis. Non-cases: remained steady	Iversen 1996
Small intestine	Diagnosis	Haemoglobin		Cases: increases at 5 year before diagnosis. Non-cases: remained steady	Edgren 2010
Stomach	Diagnosis	Haemoglobin		Cases: decline at 5 year before diagnosis. Non-cases: remained steady	Edgren 2010
Stomach	Diagnosis	Platelet count		Cases: increases at 9 months before diagnosis, with an increasing rate of change at 3 months before diagnosis	Giannakeas 2022
Thyroid	Diagnosis	Thyroid Stimulating Hormone		No difference between cases and non-cases	Rinaldi 2014

[1]Risk window is the outcome time frame. “Diagnosis” means studied analysed trends until diagnosis. Positive numbers mean the study analysed trend and predicted subsequent cancer risk, e.g. “3 years” means three-year risk following trend. Negative numbers mean the study analysed trend prior to diagnosis, e.g. “-1-2 years” means 1-2 years of trend prior to diagnosis.

Table S10: Risk of bias in the 29 studies assessing the association between blood test trend and cancer, assessed using the QUIPS tool

Article	Participation	Attrition	Prognostic factor	Outcome	Confounders	Analysis & reporting
Atkin 2020	High	Low	High	High	High	High
Boursi 2016	Low	Low	High	Moderate	Moderate	High
Chaturvedi 2010	High	High	High	Moderate	Moderate	Moderate
Edgren 2010	Moderate	Low	High	Moderate	High	Moderate
Feng 2020	High	High	High	Low	High	Low
Fuente 2019	Moderate	Low	High	Low	High	High
Furukawa 1984	High	High	High	Moderate	High	Moderate
Giannakeas 2022	High	Low	Moderate	Moderate	High	High
Goldshtein 2010	High	Low	Moderate	Moderate	Moderate	High
Gradel 2020	Moderate	Low	High	Moderate	Moderate	High
Hauser 2021	Moderate	Moderate	High	Moderate	High	Moderate
Hsieh 2019	Moderate	Low	Moderate	High	High	High
Huang 2020	Moderate	Low	Low	Low	High	Low

Iversen 1996	Moderate	High	Low	Moderate	High	High
Jacobson 2021	Moderate	High	High	Low	Low	Moderate
Jonsson 2020	Moderate	High	Low	Moderate	Moderate	Moderate
Koshiaris 2018	Low	Low	Low	Low	Low	Low
Kubo 2016	High	Low	Low	Low	High	Moderate
Lemanska 2022	Low	Low	Low	Low	Low	Low
Li 2021	Moderate	Low	Low	Low	Low	Moderate
Pannala 2009	Low	Low	Low	Low	High	High
Rinaldi 2014	Low	High	High	Low	Moderate	Low
Sadr-Azodi 2015	Moderate	Low	High	Low	Moderate	Moderate
Sharma 2018	Moderate	Low	High	Low	High	Moderate
Stroud 2020	Moderate	High	Low	Low	Moderate	Moderate
Tan 2023	Low	Low	Moderate	Low	Low	Low
Toriola 2011	High	Low	High	Low	Moderate	Moderate
Toriola 2013	High	Low	High	Low	Low	Low
Virdee 2022	Low	Low	Low	Low	Low	Low

Total low (%)	7 (24%)	20 (69%)	10 (34%)	17 (59%)	7 (24%)	8 (28%)
Total moderate (%)	13 (45%)	1 (3%)	4 (14%)	10 (34%)	9 (31%)	12 (41%)
Total high (%)	9 (31%)	8 (28%)	15 (52%)	2 (7%)	13 (45%)	9 (31%)
